# A New Pathway for the Synthesis of a New Class of Blue Fluorescent Benzofuran Derivatives

**DOI:** 10.3390/molecules23081968

**Published:** 2018-08-06

**Authors:** Costel Moldoveanu, Ionel Mangalagiu, Dragos Lucian Isac, Anton Airinei, Gheorghita Zbancioc

**Affiliations:** 1 Chemistry Department, Alexandru Ioan Cuza University of Iasi, 700506 Iasi, Romania; costel.moldoveanu@uaic.ro (C.M.); ionelm@uaic.ro (I.M.); 2 Petru Poni Institute of Macromolecular Chemistry, 700487 Iasi, Romania; dragos.isac@chem.uaic.ro (D.L.I.); airineia@icmpp.ro (A.A.)

**Keywords:** benzofuran, microwave irradiation, fluorescent, dihydroxyacetophenone

## Abstract

In this study an efficient and straightforward method for obtaining a new class of blue fluorescent bezofuran derivatives, under microwave irradiation, as well as under conventional thermal heating, is presented. Under conventional TH the reactions occur selectively, and a single type of benzofuran ester derivative was obtained. The synthesis under MW irradiation also led to benzofuran derivatives, but in a time-dependent manner. Irradiation for a short period of time led to a mixture of two types of benzofuran derivatives (**3a**–**c** and **4a**–**c),** while MW irradiation for a longer period of time led to a single type of benzofuran (3-methylbenzofuran), the reaction becoming highly selective. Taking into consideration the advantages offered by MW irradiation in terms of a substantial decrease of solvent consumed, a substantial reduction in reaction time (from days to hours), and a consequent diminution in energy consumption, these methods could be considered environmentally friendly. Here, feasible reaction mechanisms for the benzofuran derivatives formation are described. The absorption and fluorescence emission of the obtained benzofuran derivatives were studied, with part of these compounds being intense blue emitters. A certain influence of the benzofuran substituents concerning absorption and fluorescent properties was observed. Only compounds anchored with a carbomethoxy group of furan ring have shown good quantum yields.

## 1. Introduction

The synthesis of highly fluorescent derivatives with extended π-conjugation has been a topic of considerable research because of their potential applications [1,2,3,4,5,6,7,8,9]. Fused *O*-heterocyclic rings offer very interesting optical properties. Benzofuran derivatives represent such a class, being a “pure” blue-emitting moiety [1,2,3,4]. In the case of benzofuran derivatives, the literature data shows that they are chromophores with high photoluminescence and good quantum yields [2]. For this reason, benzofuran derivatives have applications in a variety of fields, including sensors and biosensors [5,6], electroluminescent materials [3,4,7], lasers, and other optoelectronic devices.

The synthesis of benzofuran derivatives has attracted special attention [8,9,10,11], thanks to their appearance in a large number of natural products [12] and synthetic pharmaceuticals [13]. Various other classes of benzofurans are attractive to chemists for their biological activities such as antimicrobial [14], antioxidant [15], anti-inflammatory [16], antifungal [17], PPARD (Peroxisome Proliferator Activated Receptor Delta) agonists [18], antifeedant, anti-HIV, anti-tumor and antiplatelet activities [19], and in other fields of chemistry and agriculture [20].

MW irradiation has become a powerful tool in organic chemistry, offering a versatile and facile pathway in a large variety of syntheses. MW-assisted synthesis has numerous advantages compared with classical synthesis: rapid energy transfer, faster reactions and substantial decreases of reaction time, fewer byproducts, improved yields and high purity of the compounds, less energy consumption, lower costs, and simplicity of handling and processing [21,22,23,24].

In previous research works [25,26], we synthesized a series of dihydroxyacetophenone (DHA) derivatives with antimicrobial and anticancer activity. The aim of this work was to find a new method of synthesis for fluorescent benzofurans, starting from DHA, to study the relationship between optical properties and structure (the effect of substituents and conjugation), and to develop a new method for the preparation of these derivatives under MW irradiation.

## 2. Results and Discussion

**Scheme 1:**
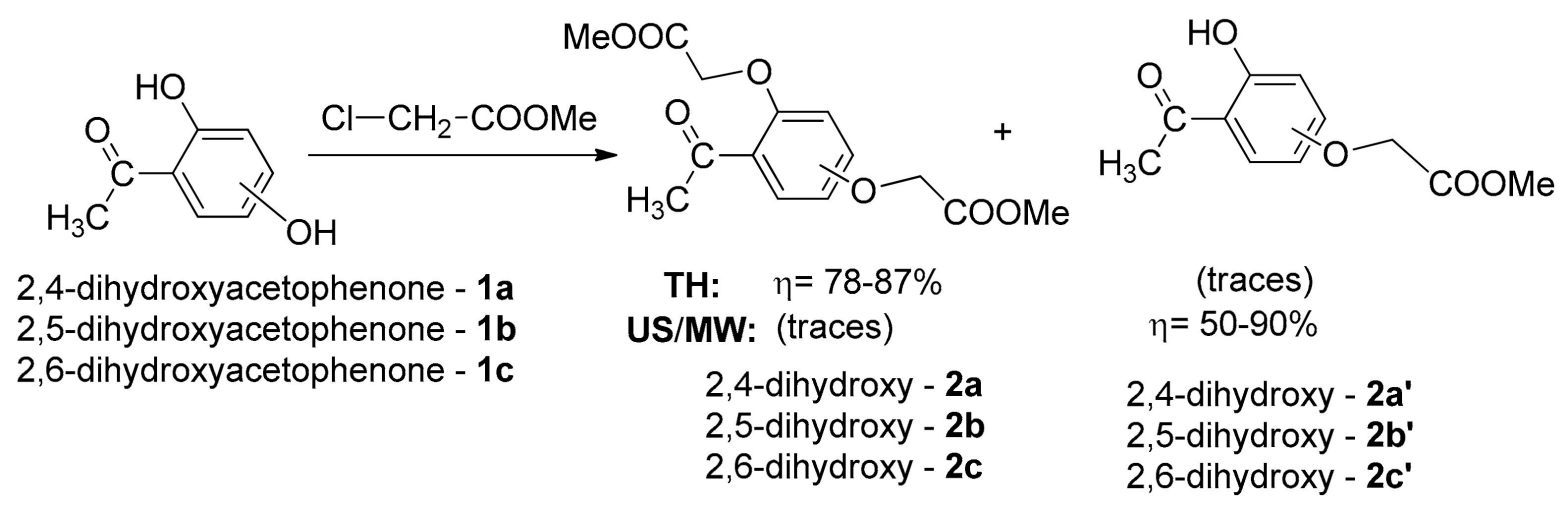
*O*-alkylation reactions of 2,n(4,5,6)-dihydroxyacetophenone derivatives.

In a continuation of our search for new entities with antimicrobial and/or anticancer activity, in previous research works [25,26] we synthesized a series of new azaheterocycle derivatives with a dihydroxyacetophenone skeleton, with the reactions being studied under conventional thermal heating (TH) and under ultrasound (US) or MW irradiation. The first stage of synthesis involved an *O*-alkylation reaction of DHA and interesting behavior was noticed in the case of DHA with a hydroxyl group at the second position of the benzene ring: a selective *bis-O*-alkylation took place under conventional TH while under US or MW irradiation a selective *mono-O*-alkylation took place (see Scheme 1).

**Scheme 2:**
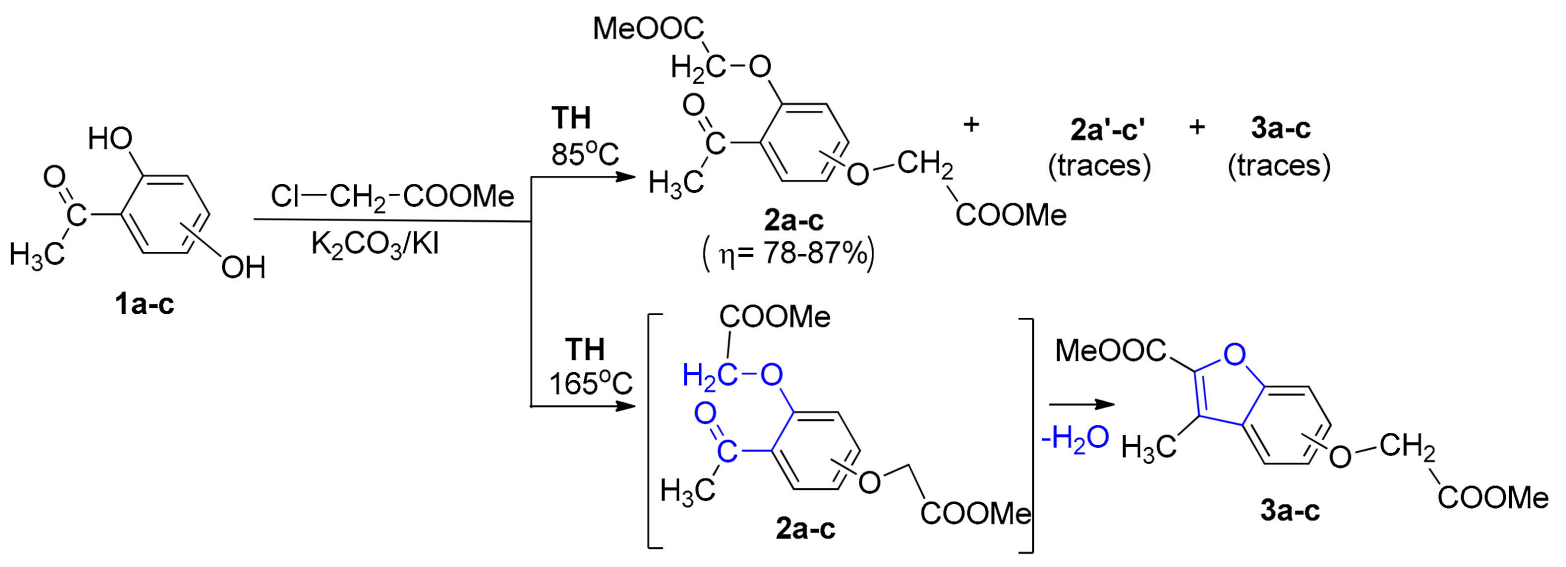
Reaction pathway for the preparation of benzofuran derivatives under thermal heating conditions.

Intrigued by these unusual behaviors, we decided to study these reactions more thoroughly. In the case of TH *bis-O*-alkylation reactions, traces of a fluorescent compound were observed besides the two types of compounds obtained (*bis*-akylated DHA **2a**–**c** and *mono*-akylated compounds **2a′**–**c′**). In order to improve the poor yields of *bis*-akylated DHA **2a**–**c**, we modified the synthesis procedure by raising the temperature. Unfortunately, the yield in *bis*-akylated DHA **2a**–**c** decreased, but, at the same time, the yield of fluorescent compound (which proved to be benzofuran ester derivative type **3**) increased substantially, and at 165 °C total conversion was observed (see Scheme 2 and Table 1).

As to the reaction mechanism, the formation of benzofuran ester derivatives could be easily explained via an intramolecular cyclocondensation of *bis*-akylated DHA **2a**–**c**, Scheme 2. This reaction mechanism is in full agreement with that previously reported by Suzuki et al. [8].

**Scheme 3:**
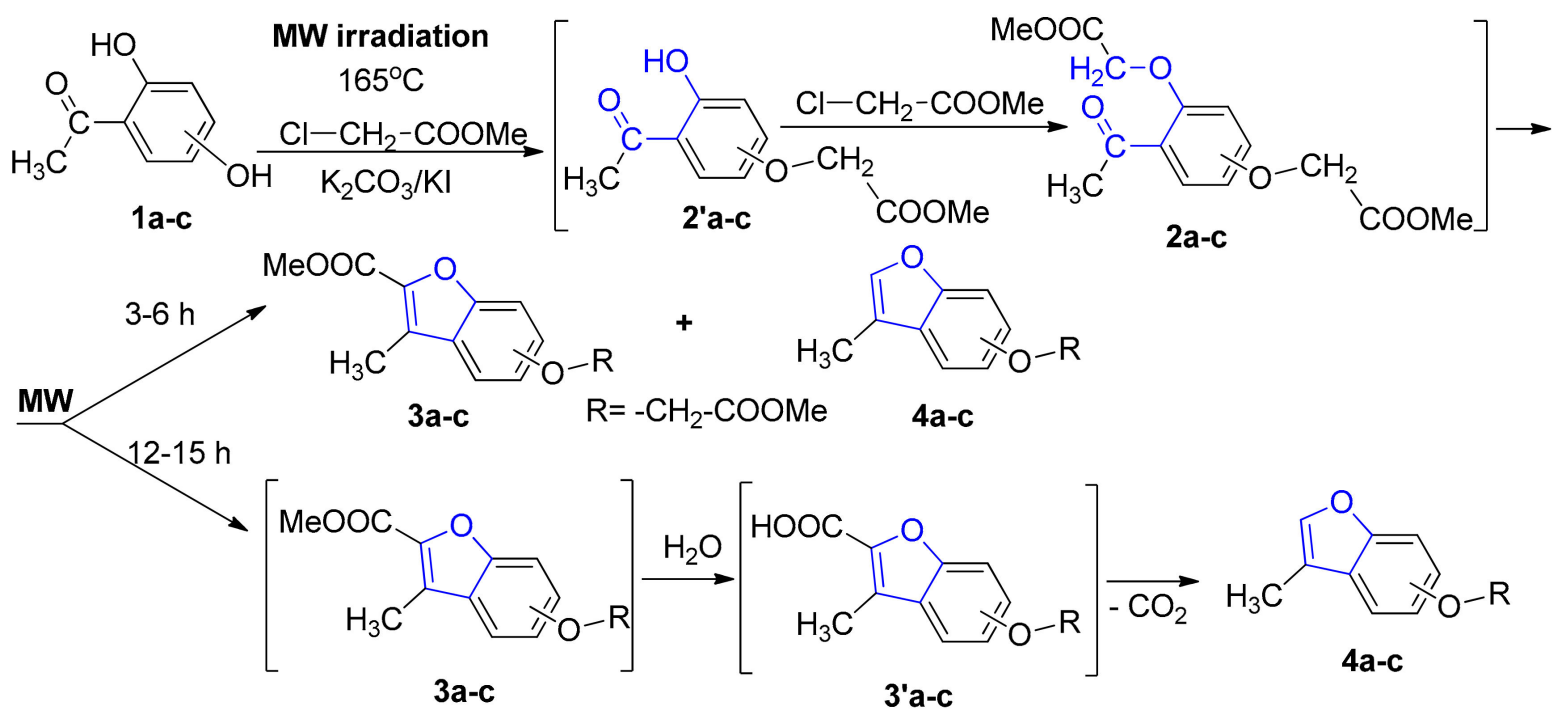
Reaction pathway for benzofuran derivatives preparation under MW irradiation.

Taking into account our expertise in the field of MW-assisted reactions in organic synthesis, we decided to perform the synthesis of the new benzofuran derivatives under MW irradiation, using a monomode reactor Monowave 300 (Anton Paar, Graz, Austria). This reactor is equipped with a stirring system (0 to 1200 rpm) and can reach up to 300 °C, with temperature control. The reactions take place in a closed vessel, at 30 bars maximum pressure. Under MW irradiation, the optimal reaction conditions were found to be at 165 °C, 17–19 bars, Scheme 3, Table 1. In contrast with conventional TH, when only one type of benzofuran derivative was obtained (**3a**–**c**), the MW-assisted reactions led either to a mixture of benzofuran derivatives, **3a**–**c** and **4a**–**c**, or to another type of benzofuran derivative (**4a**–**c**), according to the reaction time employed (see Scheme 3).

The formation of the 3-methylbenzofuran derivatives (**4a**–**c**) could be explained by the following reaction mechanism: an initial hydrolysis of the ester derivative (**3a**–**c**) led to the corresponding acids (**3′a**–**c**), which are unstable under these conditions, and undergo a subsequent oxidative decarboxylation to **4a**–**c** (see Scheme 3). This type of compound (3-methylbenzofuran derivatives) was reported to be obtained in the literature, but only as a trace [8], or in multiple steps with low overall yields [9,10,11]. Our synthetic strategy has the major advantages of good yield and easy workup (single-step reaction).

The data listed in Table 1 indicate that, when the reactions were carried out under TH conditions, a selective cyclocondensation reaction occurred, leading to the benzofuran ester derivative type **3a**–**c**, in good yields (74–79%). Under MW irradiation, according to the reaction time employed, the reactions occurred differently. When MW irradiation was performed for a short period of time (3–6 h), a mixture of benzofuran derivatives (**3a**–**c** and **4a**–**c**) was obtained in approximately equal proportions, in almost quantitative yields overall (around 95%). When MW irradiation was performed for a longer period of time (12–15 h), only one type of benzofuran derivative (3-methylbenzofurans (**4a**–**c**)) was obtained in a similar quantitative yield.

Also, it is worth mentioning that, under MW irradiation, the reaction time decreased substantially (from days to hours), the consumed energy decreased considerably, and the amount of solvent used was at least five times lower (see Section 3) compared with conventional TH. Thus, the cyclocondensation reaction could be considered environmentally friendly.

The structures of all new compounds were proven unambiguously by elemental and spectral analysis (IR, ^1^H NMR, ^13^C NMR, two-dimensional experiments 2D-COSY, 2D-HETCOR (HMQC), long-range 2D-HETCOR (HMBC).

### 2.1. UV-Vis Study

**Figure 1:**
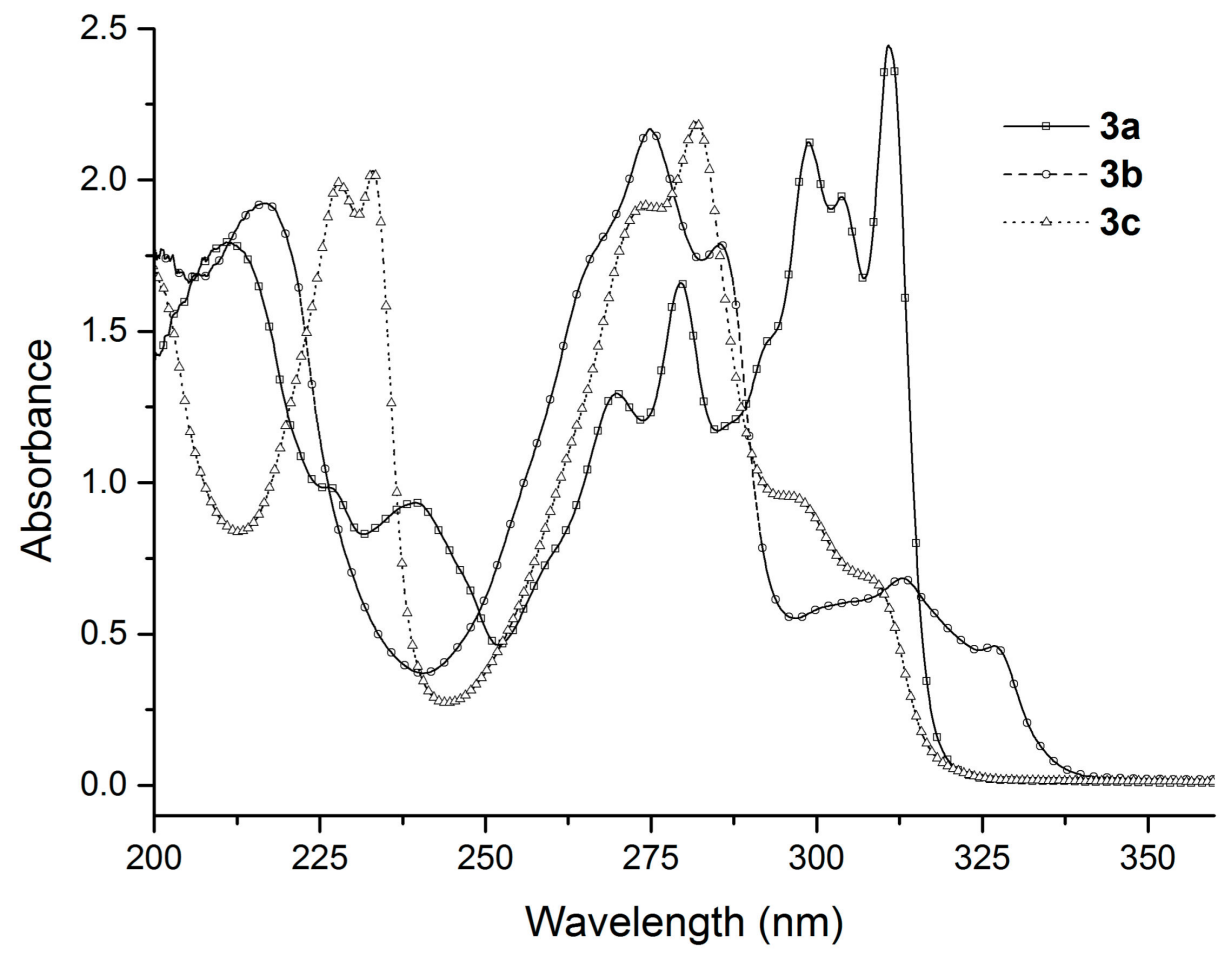
The absorption spectra of the benzofuran derivatives **3a**–**c** recorded in 10^−4^ M cyclohexane solution.

**Figure 2:**
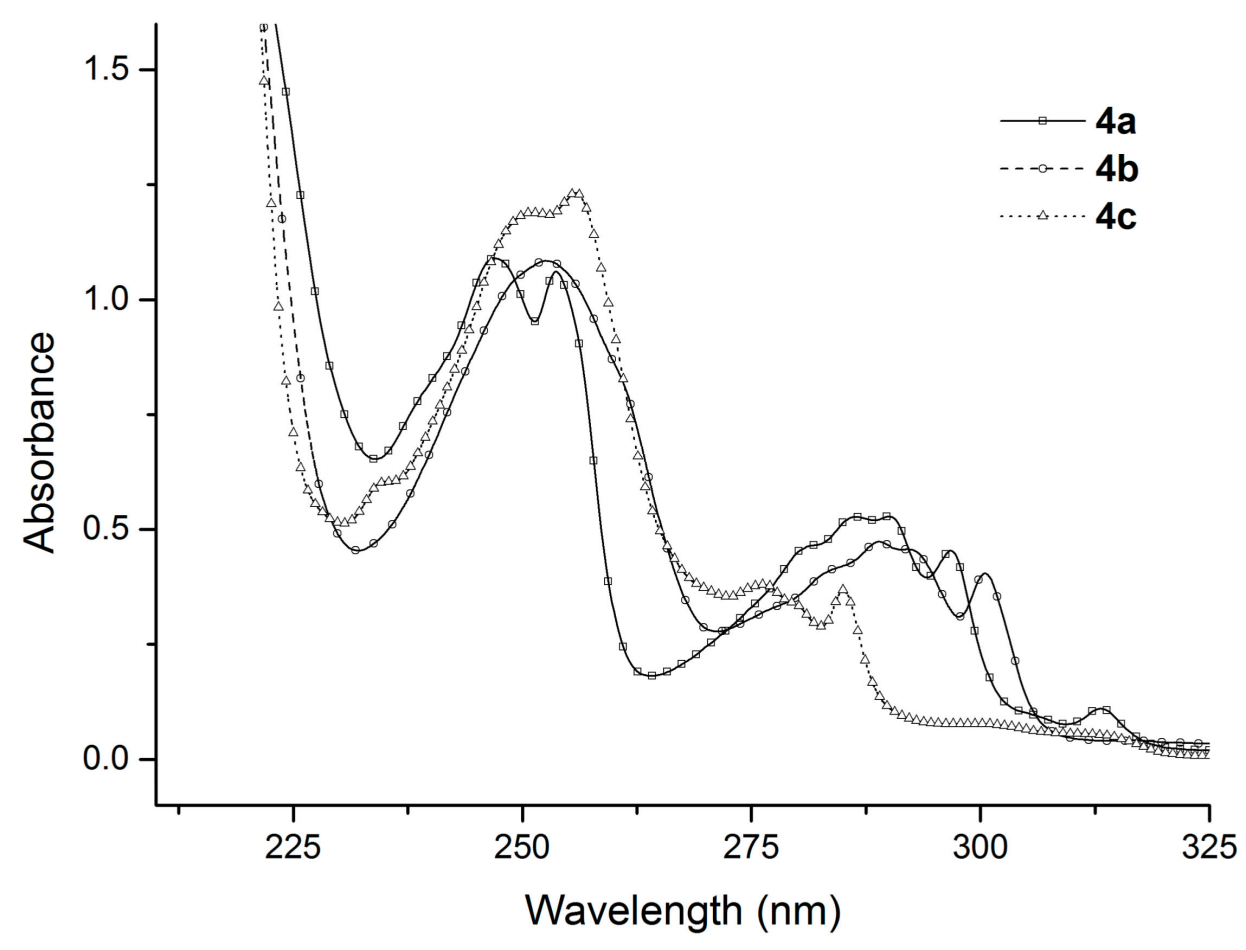
The absorption spectra of the benzofuran derivatives **4a**–**c** recorded in 10^−4^ M cyclohexane solution.

In the next stage of our work, we studied the absorption and emission spectra of the obtained benzofuran derivatives. Although compounds **3a**–**c** and **4a**–**c** have a relatively similar structure, they exhibit clear differences in their experimental UV-Vis absorption spectra, as can be seen from Figure 1 and Figure 2.

The absorption maxima of the six benzofuran derivatives in cyclohexane and the corresponding molar extinction coefficients, ε, are summarized in Table 2.

As we can observe in Table 2 and Figure 1 and Figure 2, the position of the absorption bands and the values of the molar extinction coefficients show the influence of the substituents on the benzofuran ring.

In the case of compounds **4a**–**c** (Figure 2), two well-separated absorption regions are observed (between 230–270 nm and 270–310 nm, respectively). The absorption bands from the first region have a higher (more than double) intensity than the absorption bands from the second region. Also, in the first region the absorption bands have similar intensities and profiles, while in the second region the differences between the absorption bands are higher. The maximum of the absorption of all three compounds **4a**–**c** have close values: 247.0 nm for the compound **4a**, 252.4 nm for the compound **4b**, and 255.8 nm for the compound **4c**.

In the case of compounds **3a**–**c** (Figure 1), the absorption spectral profile is quite different. In this case, two well-separated absorption regions were also observed (between 230–295 nm and 295–335 nm, respectively). For compounds **3b** and **3c**, as in the previous case, the absorption bands from the first region had a higher intensity (more than double) than the absorption bands from the second region. Regarding the maximum of the absorption of the compounds **3b** and **3c**, these appear in the first region, but in different bands. Thus, for compound **3b** the absorption maximum appeared at 274.8 nm and a secondary band appeared around 285.4 nm, while for compound **3c** the situation was reversed: the absorption maximum appeared at 281.8 nm and the secondary band appeared around 274.2 nm. Surprisingly, for compound **3a**, the absorption spectrum was totally different. The absorption bands from the first region had a smaller intensity compared to the absorption bands from the second region. Accordingly, a bathochromic shift of the absorption maximum to 310.8 nm was observed.

### 2.2. Fluorescence Study

**Figure 3:**
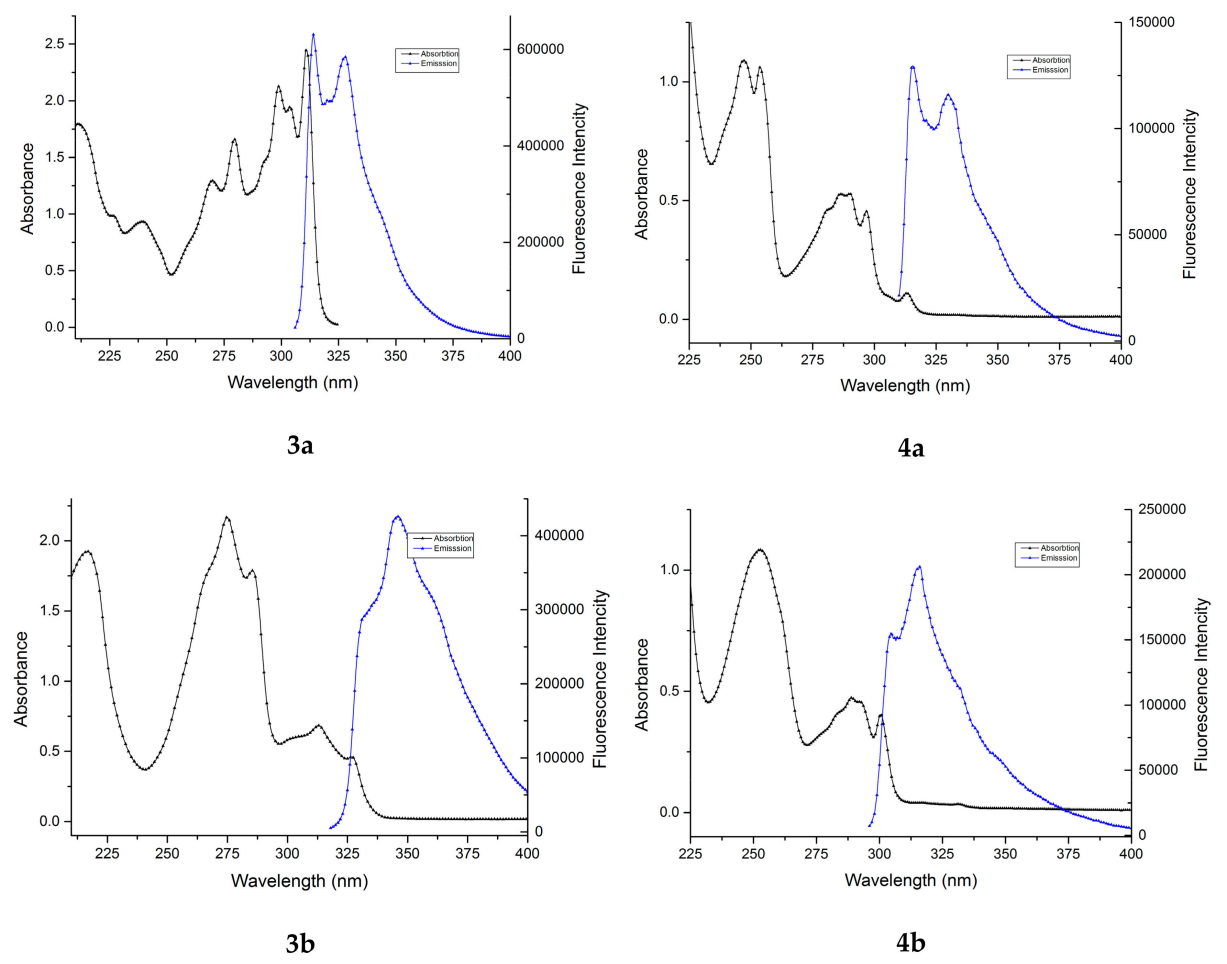

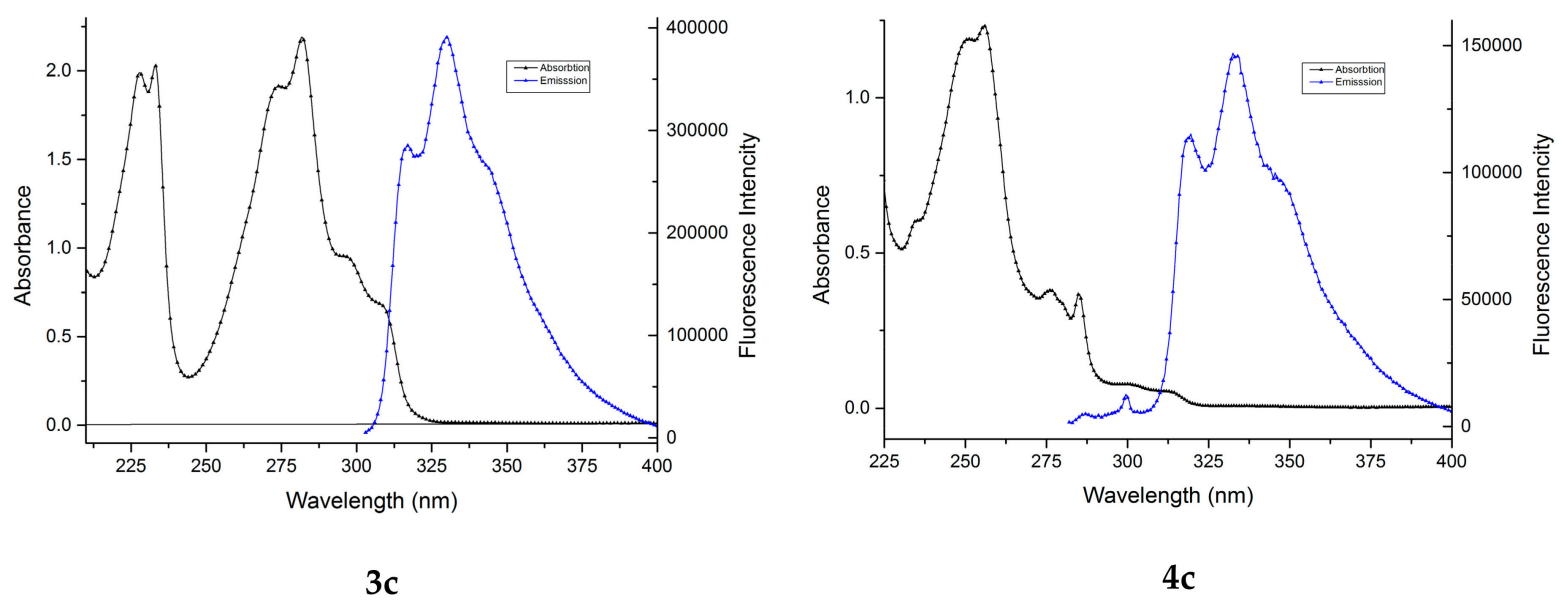
The absorption and emission spectra of benzofuran derivatives **3a**–**c** and **4a**–**c** in cyclohexane. Excitation wavelengths were: 300 nm for **3a**, 312 nm for **3b**, 297 nm for **3c**, 304 nm for **4a**, 290 nm for **4b**, and 276 nm for **4c**.

All the studied benzofuran derivatives have emission spectra consisting of one structured band in the 305–370 nm region (Figure 3), indicating a planar structure of the molecules. The position of the band is significantly influenced by the presence of a carbomethoxy group at the 2 position of benzofuran ring. In the first case, a bathochromic shift dropped of Δ_max_ = 3.5 nm (**3a** compared with **4a** and **3c** with **4c**) and in the second case a hypsochromic shift of Δ_max_ = 29.5 nm (**3b** compared with **4b**) could be observed (Table 3).

The fluorescence quantum yields of benzofuran derivatives are dramatically dependent on the presence of a carbomethoxy group in the furan ring. As seen in Table 3, compounds **3a**–**c** have a high quantum yield (41–55%), while compounds **4a**–**c** have moderate quantum yield values (17–20%). The highest fluorescence absolute quantum yield (54.85%) was observed for compound **3b**, thus making it interesting for applications as fluorophores in biolabeling and environmental trace analysis.

Figure 3 presents the absorption and emission spectra of compounds **3a**–**c** and **4a**–**c** in cyclohexane.

## 3. Experimental Section

### 3.1. General Procedure

All the reagents and solvents were purchased from commercial sources and used without further purification. Melting points were recorded on a MEL-TEMP II apparatus in open capillary tubes and are uncorrected. Analytical thin-layer chromatography was performed with commercial silica gel plates 60 F_254_ (Merck Darmstadt, Germany) and visualized with UV light. The NMR spectra were recorded on a (Bruker Vienna, Austria) Avance III 500 MHz or (Bruker Vienna, Austria) Avance DRX 400 MHz spectrometer operating at 500/400 MHz for ^1^H and 125/100 MHz for ^13^C. The following abbreviations were used to designate chemical shift multiplicities: s = singlet, d = doublet, t = triplet, m = multiplet. Chemical shifts were reported in delta (δ) units, part per million (ppm) and coupling constants (*J*) in Hz. Infrared (IR) data were recorded as films on potassium bromide (KBr) pellets on a FT-IR (Shimadzu Kyoto, Japan) Prestige 8400s spectrophotometer. The purity of the compounds was proved by HPLC on a (Thermo Scientific Massachusetts, USA) UHPLC UltiMate 3000 apparatus. For the microwave irradiation we used a monomode reactor Monowave 300 (Anton Paar Graz, Austria). This reactor can reach up to 300 °C and controls the temperature via a built-in IR sensor. Additionally, simultaneous IR and internal temperature monitoring is possible with the so-called Ruby Thermometer. Some additional specifications of Monowave 300: microwave power 850 W, operation limits at 300 °C and 30 bars, reaction vial Borosilicat, operation volume between 0.5–20 mL, pressure control by hydraulic system, agitation with an integrated magnetic stirrer (0 to 1200 rpm) and cooling with compressed air. UV-Vis spectra were recorded on a Shimadzu 1800 PC spectrophotometer in cyclohexane (spectroscopic grade) solution. The fluorescence measurements were made using a (Perkin Elmer Massachusetts, USA) LS55 luminescence spectrometer, in the same solvents as for the UV-Vis spectra, with the excitation wavelength set to the absorption band maximum. For all spectral determinations the solutions were kept in 10 mm path length quartz cells. The fluorescence quantum yield was determined at room temperature with an (Edinburgh Instruments, Edinburgh, UK FLS 980 fluorimeter with an integrating sphere and the excitation wavelength corresponds to the maximum of the absorption band. MS spectra were recorded on a (Shimadzu Kyoto, Japan) GCMS-QP 2010. The instrument was running in chemical ionization (CI) mode. The system was equipped with a 25 m × 0.25 mm × 0.25 μm DB-5ms capillary column. The ion source, quadrupole, and interface temperatures were 330 °C. Helium was used as the carrier gas at constant flow (1.54 mL/min) with an initial pressure of 90.7 kPa, while methane was used as the reagent gas in the mass spectrometer. The electron multiplier voltage was set to 1750 V. Two microliters of diluted solution were injected in cold pulsed splitless mode (initial injector temperature at 250 °C). The temperature of the DB-5ms column was programmed to go from 50 °C, where it stayed for 2 min, to 320 °C at a rate of 15 °C/min and finally stayed for 5 min at 320 °C.

#### 3.1.1. General Procedure for Synthesis of Benzofuran Derivatives **3a**–**c** under Conventional TH Conditions

To a mixture of dihydroyacetophenone derivatives (5 mmol, 0.761 g), potassium carbonate (10 mmol, 1.382 g) and potassium iodide (a spatula tip) in 100 mL acetonitrile, methylchloroacetate were added (10 mmol, 0.88 mL) at room temperature. Under conventional TH, the solution was then refluxed at 165 °C for four days on an oil bath. After the completion of the reaction (TLC), the obtained solution was cooled at room temperature; water was added and then the compounds were extracted with chloroform (3 × 50 mL), washed with a solution of sodium hydroxide (10%), dried and evaporated, with precipitate formation. The purification of the crude product was done by column chromatography on silica gel (eluted with dichloromethane).

#### 3.1.2. General Procedure for Syntheses of Benzofuran Derivatives **3a**–**c** and **4a**–**c** under Microwave Irradiation

Under MW irradiation, the mixture of reagents were dissolved in 40 mL acetonitrile, placed in the reaction vessel (borosilicate), and exposed to microwave irradiation (for 3–6 h; see Table 1). Using MW irradiation, the best results were obtained using a “temperature control” method. The “temperature control” method ensures a constant temperature (in this case 165 °C) and the power and pressure are kept constant. This method takes place in three stages. In the first step the temperature is raised as quickly as possible (within less than 1 min) by applying the maximum power. In the second step the reaction mixture is kept at a constant temperature with the control of the magnetron power. In the last step the reaction tube is cooled to 55 °C by stopping the irradiation and blowing the reaction vial with compressed air. Once the heating cycle is completed, the reaction vial is removed from the reactor, and processed as indicated for TH.

*Methyl 6-(2-methoxy-2-oxoethoxy)-3-methylbenzofuran-2-carboxylate* (**3a**). (1.06 g, 76% (under classical heating) and 0.63 g, 45% (under microwaves)) as a yellowish crystals, m.p. 134–135 °C); *R*_f_ (99.9/0.1 CH_2_Cl_2_/CH_3_OH) 0.61; **IR** (cm^−1^): 3096, 3073, 3030 (C-H arom.), 2955, 2920 (C-H aliph.), 1768, 1718 (C=O, ester), 1618, 1595, 1576, 1443, 1425, 1368 (aromatic and heteroaromatic ring), 1271, 1219, 1169, 1148, 1128, 1097, 1078 (C–O–C, ester); **^1^H NMR** (400 MHz, CDCl_3_): δ 7.51 (1H, d, *J* = 8.8 Hz, H-4), 7.00 (2H, m, overlapped peaks, H-5, H-7), 4.69 (2H, CH_2_ of methyl acetate group from 6 position, s), 3.96 (3H, CH_3_ of methoxycarbonyl group from 2 position, s), 3.82 (3H, CH_3_ of methyl acetate group from 6 position, s), 2.55 (3H, CH_3_ group from 3 position, s); **^13^C NMR** (100 MHz, CDCl_3_): δ 168.8 (CO keto ester from 6 position), 160.7 (CO keto ester from 2 position), 158.7 (C-7a), 155.3 (C-6), 140.4 (C-2), 126.2 (C-3), 123.3 (C-3a), 121.7 (C-4), 113.4 (C-5), 96.7 (C-7), 65.6 (CH_2_ of methyl acetate group from 6 position), 52.3 (CH_3_ of methyl acetate group from 6 position), 51.8 (CH_3_ of methoxycarbonyl group from 2 position), 9.3 (CH_3_ group from 3 position); **MS (CI, *m*/*z*):** 280 (M + 2; 7.5%), 279 (M + 1; 45.3%), 278 (M^+^, base peak, 100%), 247 (M–OCH_3_; 18.9%), 219 (M–COOCH_3_; 28.3%), 205 (M–CH_2_COOCH_3_; 26.4%).

*Methyl 5-(2-methoxy-2-oxoethoxy)-3-methylbenzofuran-2-carboxylate* (**3b**). (1.10 g, 79% (under classical heating) and 0.68 g, 49% (under microwaves)) as a yellowish crystals, m.p. 124–125 °C); *R*_f_ (99.9/0.1 CH_2_Cl_2_/CH_3_OH) 0.57; **IR** (cm^−1^): 3105, 3084, 3010 (C-H arom.), 2957, 2920 (C-H aliph.), 1759, 1707 (C=O, ester), 1615, 1581, 1473, 1437, 1369 (aromatic and heteroaromatic ring), 1300, 1223, 1207, 1155, 1082 (C–O–C, ester); **^1^H NMR** (400 MHz, CDCl_3_): δ 7.45 (1H, d, *J* = 9.2 Hz, H-7), 7.13 (1H, dd, *J* = 9.2, 2.4 Hz, H-6), 7.01 (1H, d, *J* = 2.4 Hz, H-4), 4.70 (2H, CH_2_ of methyl acetate group from 5 position, s), 3.98 (3H, CH_3_ of methoxycarbonyl group from 2 position, s), 3.83 (3H, CH_3_ of methyl acetate group from 5 position, s), 2.55 (3H, CH_3_ group from 3 position, s); **^13^C NMR** (100 MHz, CDCl_3_): δ 169.5 (CO keto ester from 5 position), 160.9 (CO keto ester from 2 position), 154.6 (C-5), 150.1 (C-7a), 141.8 (C-2), 129.6 (C-3a), 125.9 (C-3), 118.0 (C-6), 113.2 (C-7), 104.4 (C-4), 66.4 (CH_2_ of methyl acetate group from 5 position), 52.4 (CH_3_ of methyl acetate group from 5 position), 52.1 (CH_3_ of methoxycarbonyl group from 2 position), 9.5 (CH_3_ group from 3 position); **MS (CI, *m*/*z*):** 280 (M + 2; 5.7%), 279 (M + 1; 47.2%), 278 (M^+^, base peak, 100%), 247 (M–OCH_3_; 17.0%), 219 (M–COOCH_3_; 26.4%), 205 (M–CH_2_COOCH_3_; 17.0%).

*Methyl 4-(2-methoxy-2-oxoethoxy)-3-methylbenzofuran-2-carboxylate (***3c**). (1.03 g, 74% (under classical heating) and 0.64 g, 46% (under microwaves)) as a yellowish crystals, m.p. 108–109 °C); *R*_f_ (99.9/0.1 CH_2_Cl_2_/CH_3_OH) 0.68; **IR** (cm^−1^): 3080, 3051, 3011 (C-H arom.), 2957, 2918 (C-H aliph.), 1769, 1707 (C=O, ester), 1611, 1589, 1502, 1435, 1360 (aromatic and heteroaromatic ring), 1277, 1217, 1147, 1111 (C–O–C, ester); **^1^H NMR** (400 MHz, CDCl_3_): δ 7.30 (1H, t, *J* = 8.4, 8.0 Hz, H-6), 7.15 (1H, d, *J* = 8.4 Hz, H-7), 6.50 (1H, d, *J* = 8.0 Hz, H-5), 4.74 (2H, CH_2_ of methyl acetate group from 4 position, s), 3.97 (3H, CH_3_ of methoxycarbonyl group from 2 position, s), 3.82 (3H, CH_3_ of methyl acetate group from 4 position, s), 2.79 (3H, CH_3_ group from 3 position, s); **^13^C NMR** (100 MHz, CDCl_3_): δ 168.9 (CO keto ester from 4 position), 160.9 (CO keto ester from 2 position), 155.8 (C-7a), 154.43 (C-4), 139.9 (C-2), 128.7 (C-6), 127.00 (C-3), 119.2 (C-3a), 106.2 (C-7), 104.1 (C-5), 65.4 (CH_2_ of methyl acetate group from 4 position), 52.4 (CH_3_ of methyl acetate group from 4 position), 52.0 (CH_3_ of methoxycarbonyl group from 2 position), 11.3 (CH_3_ group from 3 position); **MS (CI, *m*/*z*):** 280 (M + 2; 3.8%), 279 (M + 1; 26.4%), 278 (M^+^, base peak, 100%), 247 (M–OCH_3_; 11.3%), 219 (M–COOCH_3_; 13.2%), 205 (M–CH_2_COOCH_3_; 18.9%).

*Methyl 2-(3-methylbenzofuran-6-yloxy)acetate* (**4a**). (0.00 g, 0% (under classical heating) and 0.54 g, 49% (under microwaves)) as a yellowish crystals, m.p. 73–74 °C); *R*_f_ (99.9/0.1 CH_2_Cl_2_/CH_3_OH) 0.84; **IR** (cm^−1^): 3117, 3080, 3009 (C-H arom.), 2951, 2922 (C-H aliph.), 1753 (C=O, ester), 1627, 1591, 1491, 1437, 1394 (aromatic and heteroaromatic ring), 1267, 1219, 1152, 1086, 1057 (C–O–C, ester); **^1^H NMR** (500 MHz, CDCl_3_): δ 7.39 (1H, d, *J* = 8.0 Hz, H-4), 7.33 (1H, d, *J* = 1.0 Hz, H-2), 6.97 (1H, d, *J* = 2.0 Hz, H-7), 6.92 (1H, dd, *J* = 8.0, 2.0 Hz, H-5), 4.67 (2H, CH_2_ of methyl acetate group from 6 position, s), 3.81 (3H, CH_3_ of methyl acetate group from 6 position, s), 2.20 (3H, CH_3_ group from 3 position, d, *J* = 1.0 Hz); **^13^C NMR** (125 MHz, CDCl_3_): δ 169.5 (CO keto ester from 6 position), 156.1 (C-7a), 155.9 (C-6), 141.0 (C-2), 123.6 (C-3), 119.8 (C-4), 115.6 (C-3a), 111.6 (C-5), 97.4 (C-7), 66.1 (CH_2_ of methyl acetate group from 6 position), 52.3 (CH_3_ of methyl acetate group from 6 position), 7.9 (CH_3_ group from 3 position); **MS (CI, *m*/*z*):** 221 (M+1; 13.7%), 220 (M^+^, base peak, 100%), 161 (M–COOCH_3_; 11.8%), 147 (M–CH_2_COOCH_3_; 47.1%).

*Methyl 2-(3-methylbenzofuran-5-yloxy)acetate* (**4b**). (0.00 g, 0% (under classical heating) and 0.51 g, 46% (under microwaves)) as a yellowish liquid); *R*_f_ (99.9/0.1 CH_2_Cl_2_/CH_3_OH) 0.82; **IR** (cm^−1^): 3117, 3073, 3010 (C-H arom.), 2957, 2926 (C-H aliph.), 1751 (C=O, ester), 1604, 1479, 1452, 1440 (aromatic and heteroaromatic ring), 1319, 1287, 1263, 1207, 1180, 1089 (C–O–C, ester); **^1^H NMR** (500 MHz, CDCl_3_): δ 7.38 (1H, d, *J* = 1.5 Hz, H-2), 7.35 (1H, d, *J* = 9.0 Hz, H-7), 6.98 (1H, d, *J* = 2.5 Hz, H-4), 6.94 (1H, dd, *J* = 9.0, 2.5 Hz, H-6), 4.69 (2H, CH_2_ of methyl acetate group from 5 position, s), 3.82 (3H, CH_3_ of methyl acetate group from 5 position, s), 2.20 (3H, CH_3_ group from 3 position, d, *J* = 1.5 Hz); **^13^C NMR** (125 MHz, CDCl_3_): δ 169.8 (CO keto ester from 5 position), 154.1 (C-5), 150.9 (C-7a), 142.7 (C-2), 129.7 (C-3a), 115.8 (C-3), 113.3 (C-6), 112.0 (C-7), 103.9 (C-4), 66.6 (CH_2_ of methyl acetate group from 5 position), 52.3 (CH_3_ of methyl acetate group from 5 position), 8.0 (CH_3_ group from 3 position); **MS (CI, *m*/*z*):** 221 (M + 1; 13.7%), 220 (M^+^, base peak, 100%), 161 (M–COOCH_3_; 19.6%), 147 (M–CH_2_COOCH_3_; 35.3%).

*Methyl 2-(3-methylbenzofuran-4-yloxy)acetate* (**4c**). (0.00 g, 0% (under classical heating) and 0.50 g, 45% (under microwaves)) as a yellowish liquid); *R*_f_ (99.9/0.1 CH_2_Cl_2_/CH_3_OH) 0.88; **IR** (cm^−1^): 3117, 3078, 3036 (C-H arom.), 2955, 2930 (C-H aliph.), 1763 (C=O, ester), 1609, 1495, 1437, 1360 (aromatic and heteroaromatic ring), 1292, 1259, 1213, 1171, 1115, 1092 (C–O–C, ester); **^1^H NMR** (500 MHz, CDCl_3_): δ 7.24 (1H, s, H-2), 7.09 (2H, m, overlapped peaks, H-6, H-7), 6.43 (1H, d, *J* = 7.0 Hz, H-5), 4.66 (2H, CH_2_ of methyl acetate group from 4 position, s), 3.76 (3H, CH_3_ of methyl acetate group from 4 position, s), 2.39 (3H, CH_3_ group from 3 position, s); **^13^C NMR** (125 MHz, CDCl_3_): δ 169.2 (CO keto ester from 4 position), 156.9 (C-7a), 153.2 (C-4), 140.3 (C-2), 124.6 (C-6), 118.7 (C-3), 115.9 (C-3a), 105.6 (C-7), 103.7 (C-5), 65.4 (CH_2_ of methyl acetate group from 4 position), 52.1 (CH_3_ of methyl acetate group from 4 position), 9.8 (CH_3_ group from 3 position); **MS (CI, *m*/*z*):** 221 (M + 1; 13.7%), 220 (M^+^, base peak, 100%), 185 (M–OCH_3_; 9.8%),161 (M–COOCH_3_; 35.3%), 147 (M–CH_2_COOCH_3_; 58.8%).

## 4. Conclusions

In conclusion, we report herein an efficient and straightforward pathway for obtaining a new class of blue fluorescent bezofuran derivatives, under microwave irradiation as well as under conventional thermal heating. Under conventional TH the reactions occur selectively, and a single type of benzofuran ester derivatives (**3a**–**c**) is obtained. Under MW irradiation, the reactions occur in different manners. When MW irradiation was performed for a short period of time, a mixture of benzofuran derivatives (**3a**–**c** and **4a**–**c**) were obtained, while when MW irradiation was performed for a longer period of time, the reaction became highly selective, and only one type of benzofuran derivative corresponding to 3-methylbenzofuran (**4a**–**c**) was obtained. Taking into consideration the advantages offered by MW irradiation in terms of yield, easy workup of the reaction, substantial decrease in solvent consumed (several-fold less compared with conventional TH), substantial reduction in reaction time (from days to hours) and consequent diminution in energy consumption, these methods could be considered environmentally friendly. Feasible reaction mechanisms for the benzofuran derivatives formation are described fully in this study and are in agreement with the literature. The absorption and fluorescence emission of the obtained benzofuran derivatives were studied, with part of these compounds being intense blue emitters. The fluorescence quantum yields of benzofuran derivatives are dramatically dependent on their structure, with only compounds anchored by a carbomethoxy group of furan ring having good quantum yields.

## Figures and Tables

**Table 1:** The optimized conditions for synthesis of benzofuran derivatives (**3a**–**c** or/and **4a**–**c**), under MW and TH conditions.

Compound	Microwaves			Conventional TH		
	Reaction Time, (Hours)	Temperature, °C	Yield, %	Reaction Time, (Hours)	Temperature, °C	Yield, %
**3a**	5	165	45	96	165	76
**3b**	6	165	49	96	165	79
**3c**	3	165	46	96	165	74
**4a**	5	165	49	96	165	0
	13		88			
**4b**	6	165	46	96	165	0
	15		85			
**4c**	3	165	45	96	165	0
	12		80			

**Table 2:** Absorption (λ_abs_ and ε) for the benzofuran derivatives **3a**–**c** and **4a**–**c** in cyclohexane.

Compound											
3a		3b		3c		4a		4b		4c	
λ_abs_	ε × 10^3^	λ_abs_	ε × 10^3^	λ_abs_	ε × 10^3^	λ_abs_	ε × 10^3^	λ_abs_	ε × 10^3^	λ_abs_	ε × 10^3^
211.6	17.93	216.6	19.23	227.8	19.90	**247.0**	**10.89**	**252.4**	**10.84**	235.2	6.04
226.0	9.84	**274.8**	**21.66**	233.0	20.31	252.8	10.27	288.8	4.73	251.2	11.89
239.4	9.33	285.4	17.87	274.2	19.15	286.4	5.27	292.0	4.57	**255.8**	**12.32**
269.8	12.95	313.0	6.83	**281.8**	**21.91**	290.0	5.29	300.4	4.04	276.2	3.80
279.4	16.60	326.8	4.60	295.6	9.55	296.6	4.54			285.0	3.68
298.8	21.26					313.0	1.10				
303.8	19.44										
**310.8**	**24.46**										

λ_abs_ (nm); ε × 10^3^ (L mol^−1^ cm^−1^).

**Table 3:** Fluorescence parameters: emission maximum wavelength, λ_mem_ and fluorescence quantum yield, of compounds **3a**–**c** and **4a**–**c** in cyclohexane.

Compound	*λ*_mem_ (nm)	Quantum Yields (%)
3a	314.0	49.61
3b	345.5	54.85
3c	330.0	41.58
4a	315.5	18.98
4b	316.0	20.22
4c	333.5	17.18

## Supplementary Materials

Details of the IR spectra, NMR spectra (^1^H NMR and ^13^C NMR) and HPLC chromatograms of the synthesized compounds can be found in the Supporting Information. Supplementary material is available online at http://www.mdpi.com/1420-3049/23/8/1968/s1, on the publisher’s web site.
