# Supplementary material for: A New Pathway for the Synthesis of a New Class of Blue Fluorescent Benzofuran Derivatives
Source: Molecules. 2018 Aug 6;23(8):1968. doi: 10.3390/molecules23081968 (PMC6222448; doi:10.3390/molecules23081968)
Supplement: Supplementary file 1 [file molecules-23-01968-s001.pdf]

Supporting information for:

# A New Pathway for the Synthesis of a New Class of Blue Fluorescent Benzofuran Derivatives

**Costel Moldoveanu [1], Ionel Mangalagiu [1], Dragos Lucian Isac [2], Anton Airinei [2] and Gheorghita Zbancioc [1,*]**

[1] Chemistry Department, Alexandru Ioan Cuza University of Iasi, 11 Carol 1st Bvd, Iasi -700506, Romania; costel.moldoveanu@uaic.ro (C.M.); ionelm@uaic.ro (I.M.)
[2] Petru Poni Institute of Macromolecular Chemistry,41A Grigore Ghica Voda Alley, Iasi-700487, Romania; dragos.isac@chem.uaic.ro (D.L.I.); airineia@icmpp.ro (A.A.)
* Correspondence: gheorghita.zbancioc@uaic.ro; Tel.: +40-232-201278

## Contents

1. HPLC chromatograms of the obtained compounds. .......... 2
2. NMR Spectra of the obtained compounds. .......... 5
3. IR Spectra of the obtained compounds. .......... 11

# 1. HPLC chromatograms of the obtained compounds.

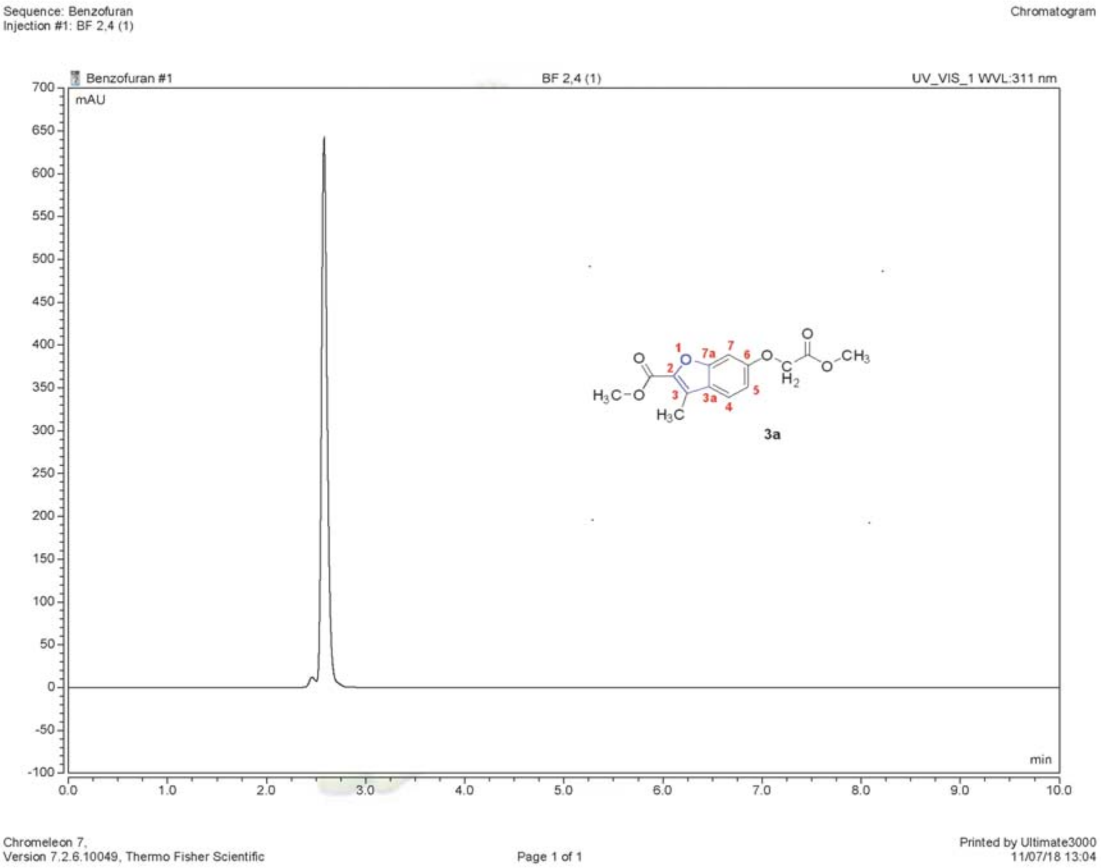


**S1 Fig.** HPLC chromatogram of the compound **3a.**

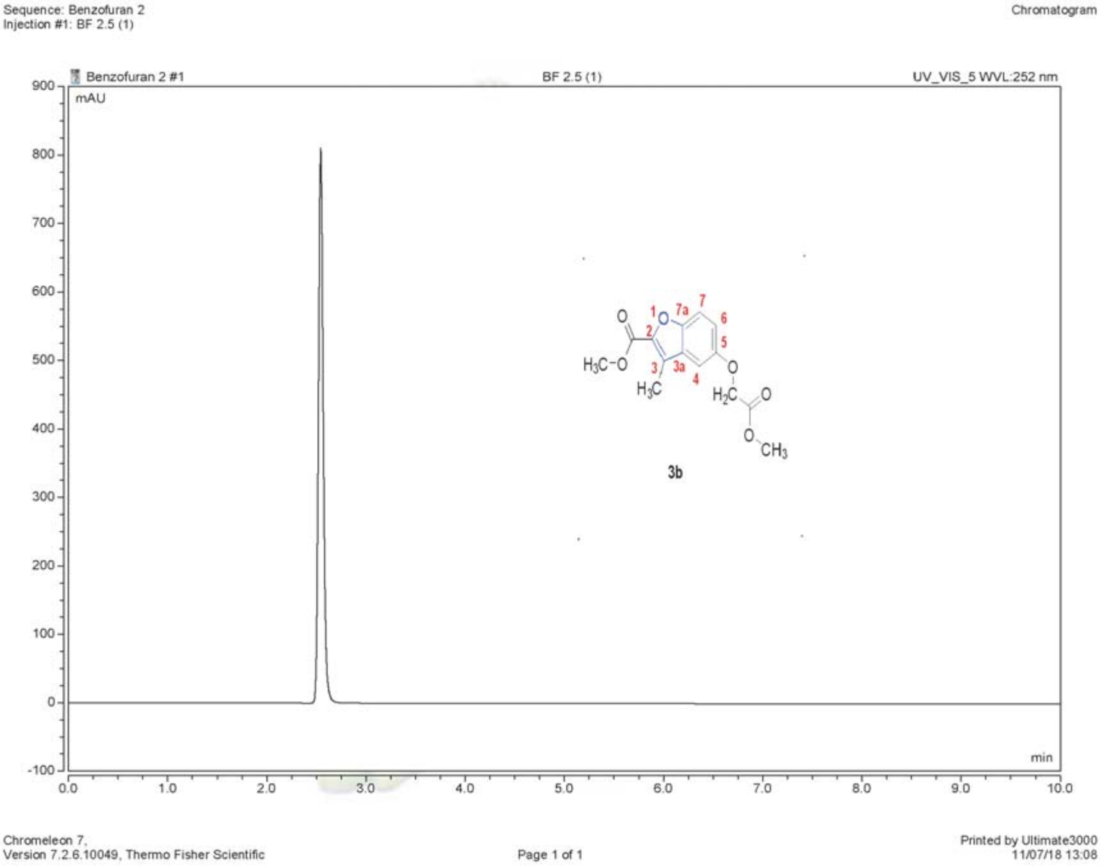


**S2 Fig.** HPLC chromatogram of the compound **3b.**

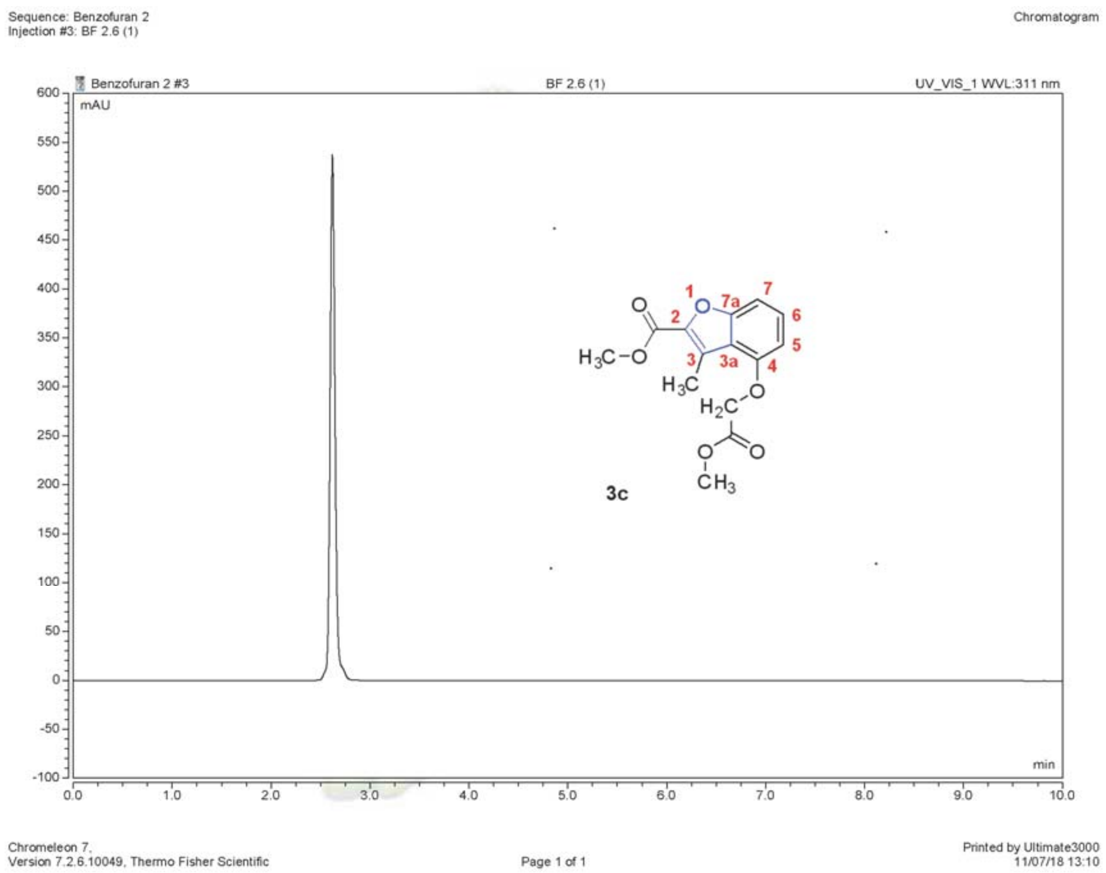


**S3 Fig.** HPLC chromatogram of the compound **3c.**

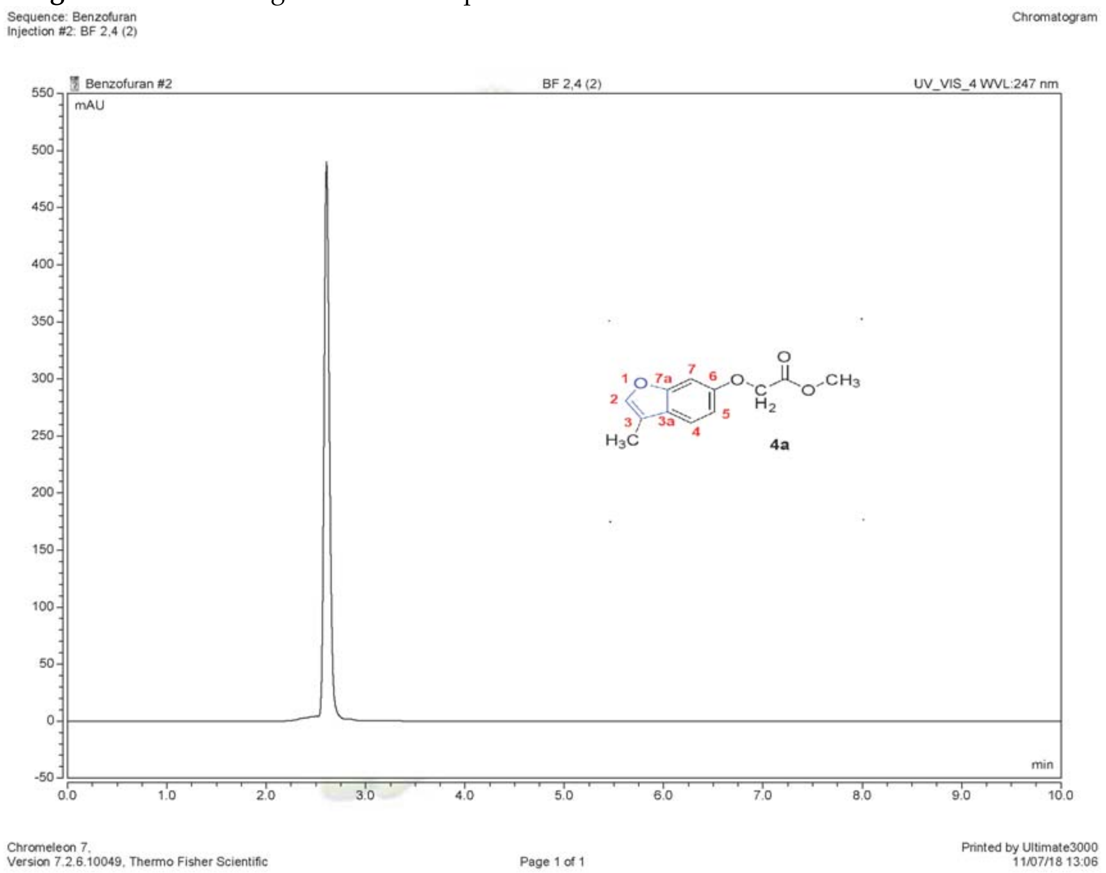


**S4 Fig.** HPLC chromatogram of the compound **4a.**

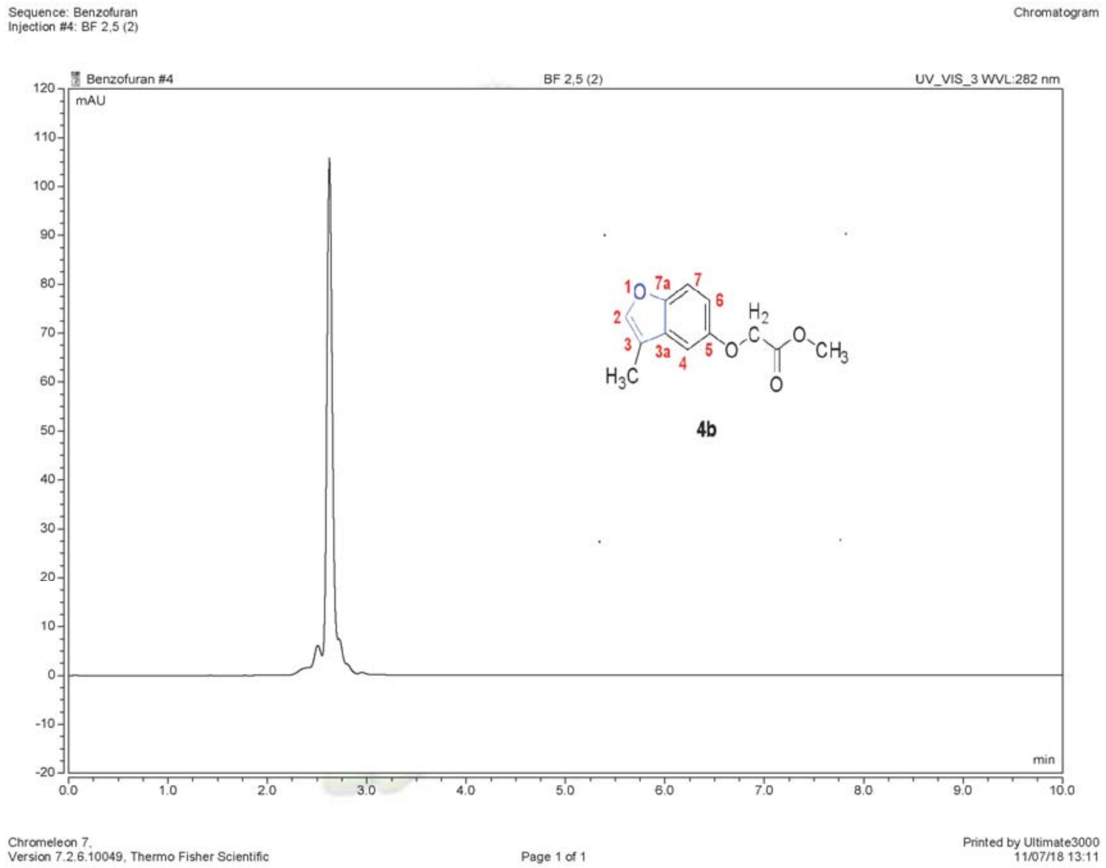


**S5 Fig.** HPLC chromatogram of the compound **4b.**

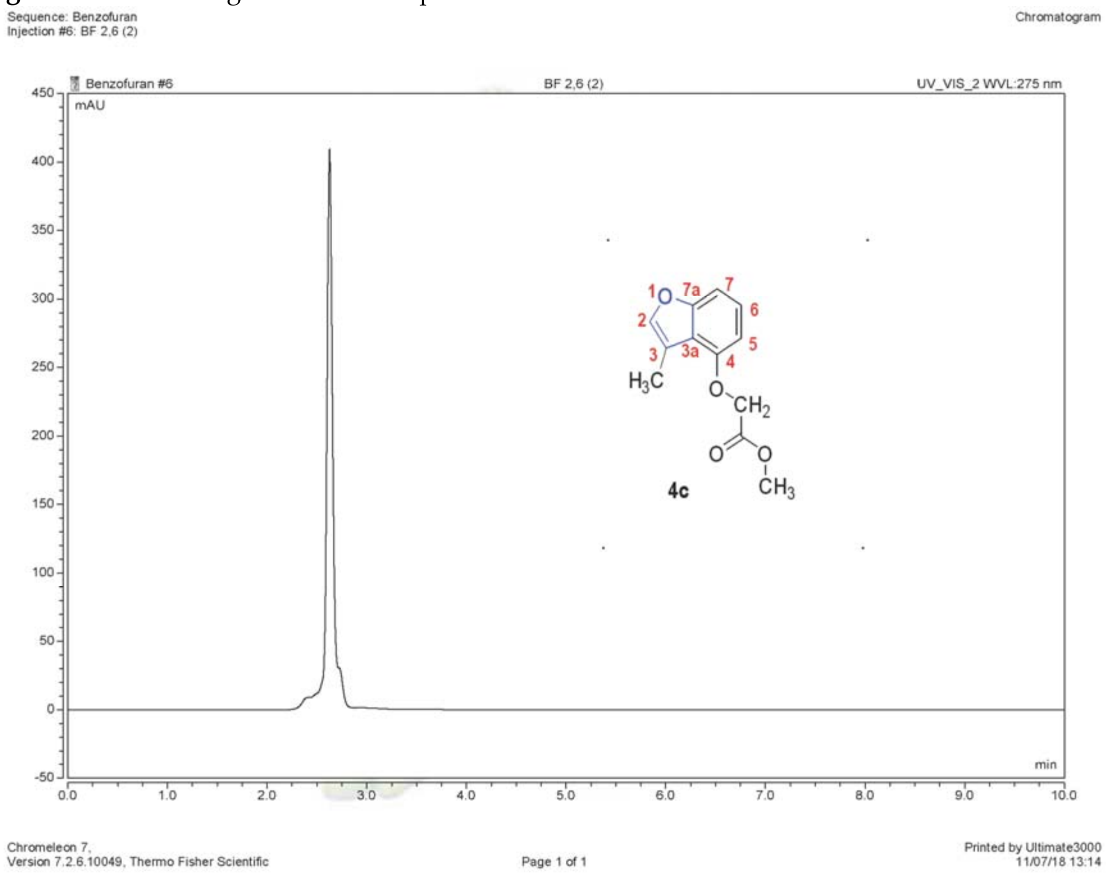


**S6 Fig.** HPLC chromatogram of the compound **4c.**

## 2. NMR Spectra of the obtained compounds.

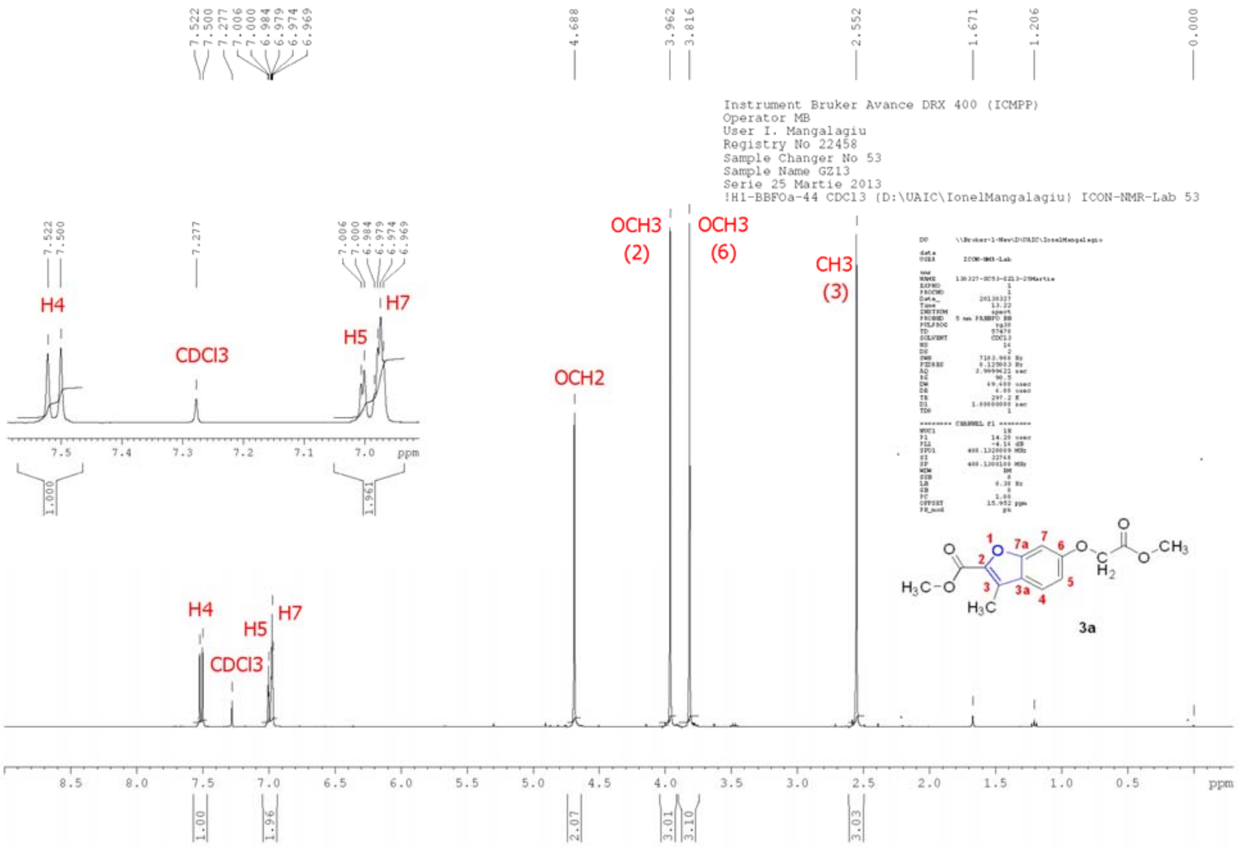


**S7a Fig.** $^{1}$H NMR spectrum of the compound **3a.**

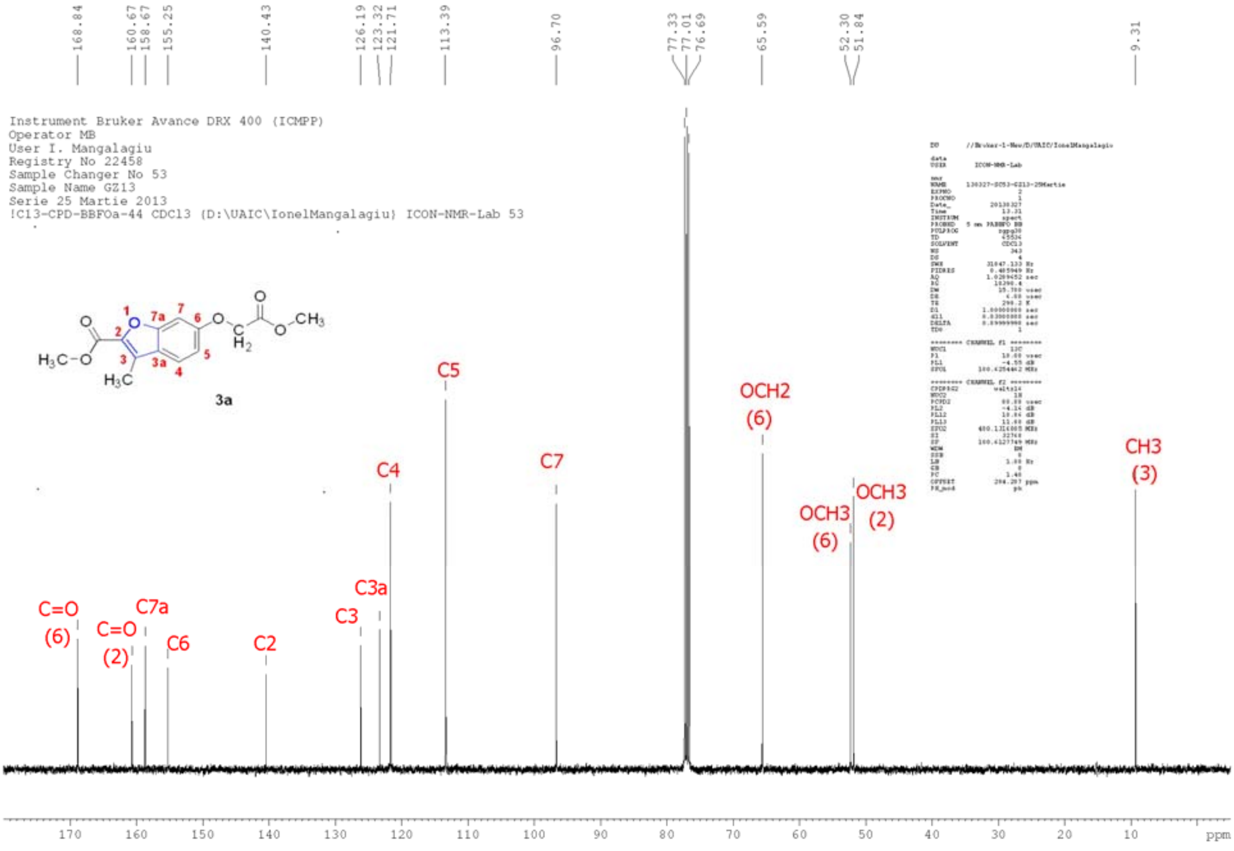


**S7b Fig.** $^{13}$C NMR spectrum of the compound **3a.**

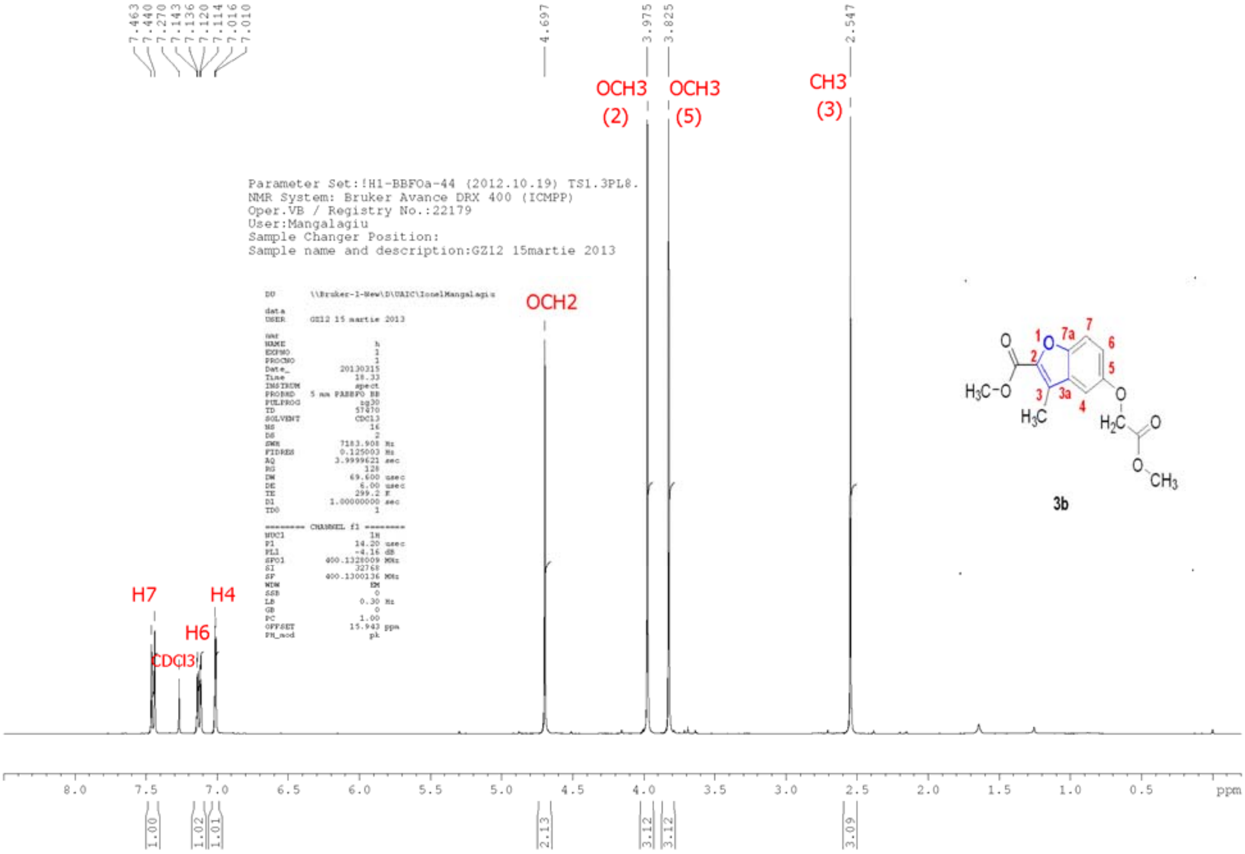


**S8a Fig.** $^{1}$H NMR spectrum of the compound **3b.**

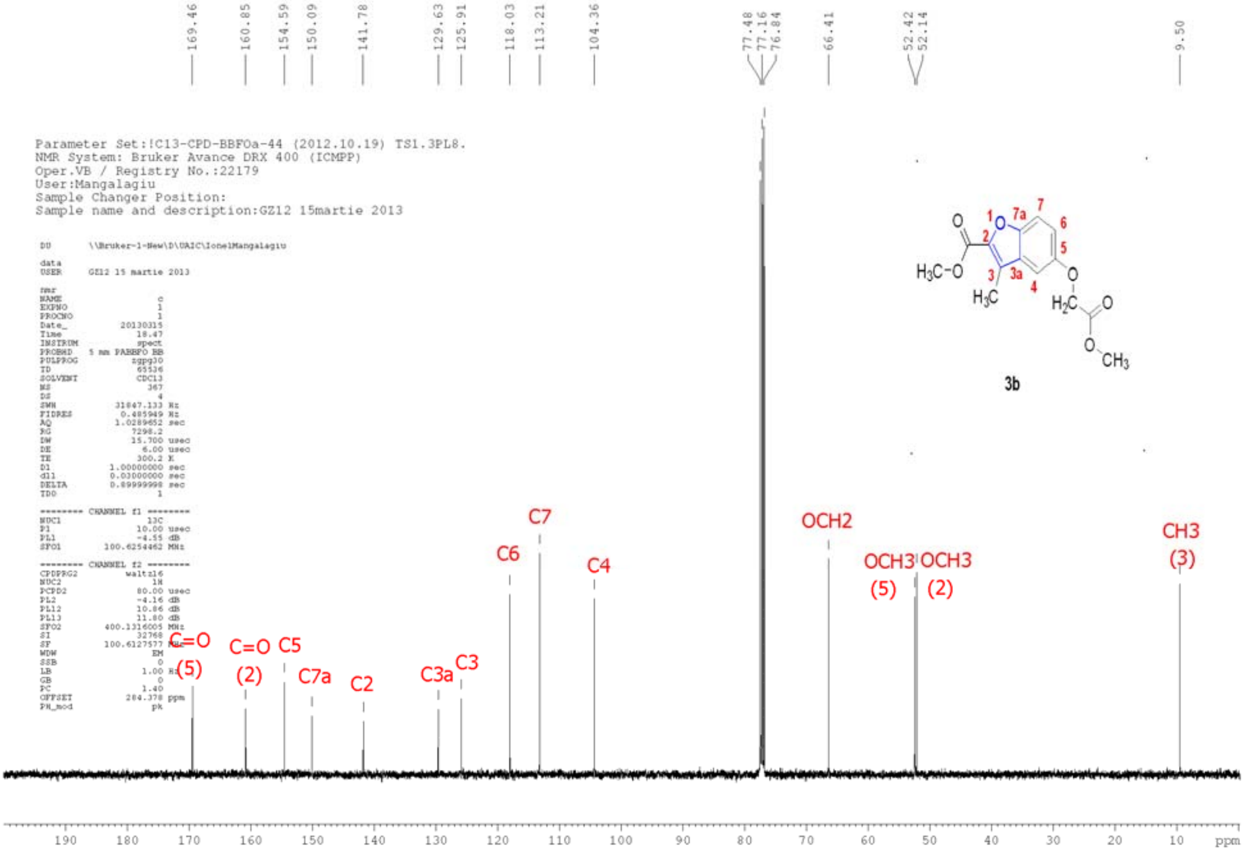


**S8b Fig.** $^{13}$C NMR spectrum of the compound **3b.**

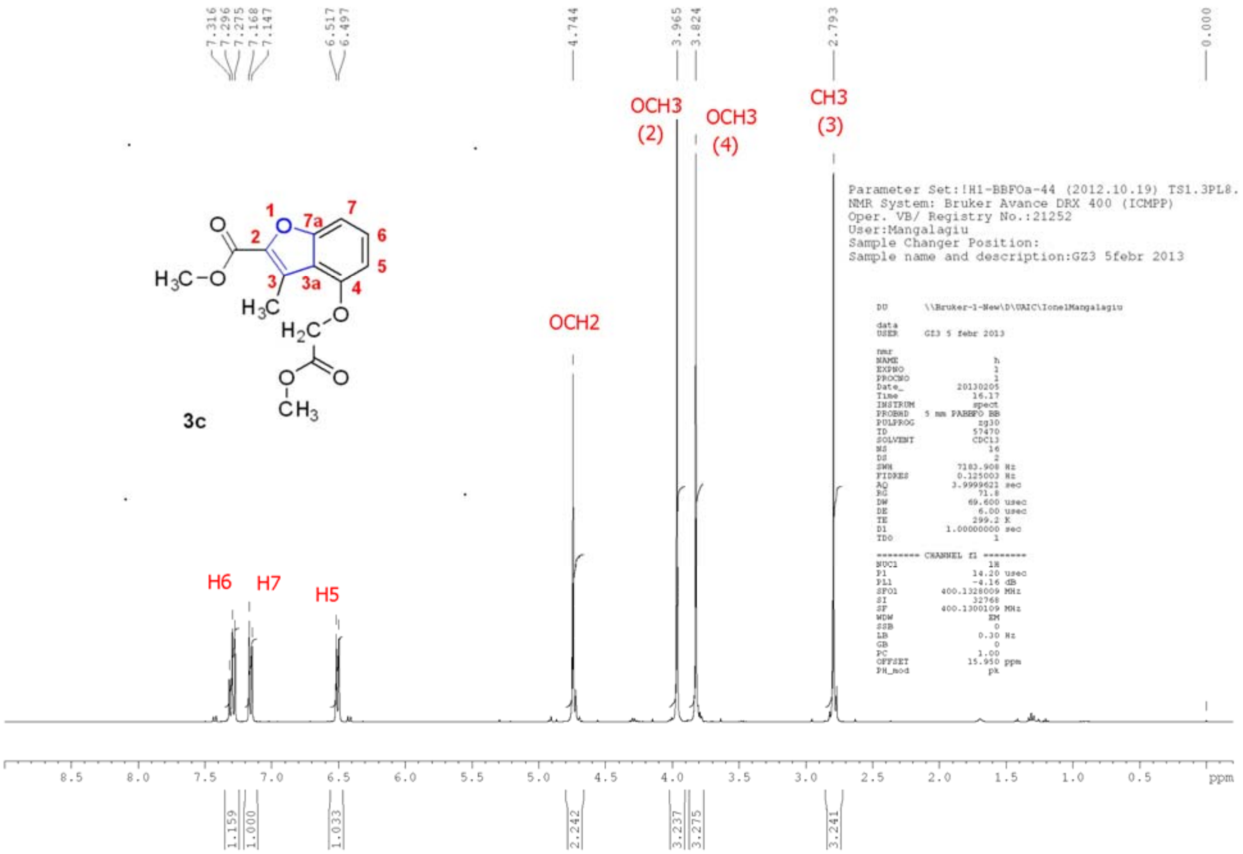


**S9a Fig.** $^{1}$H NMR spectrum of the compound **3c.**

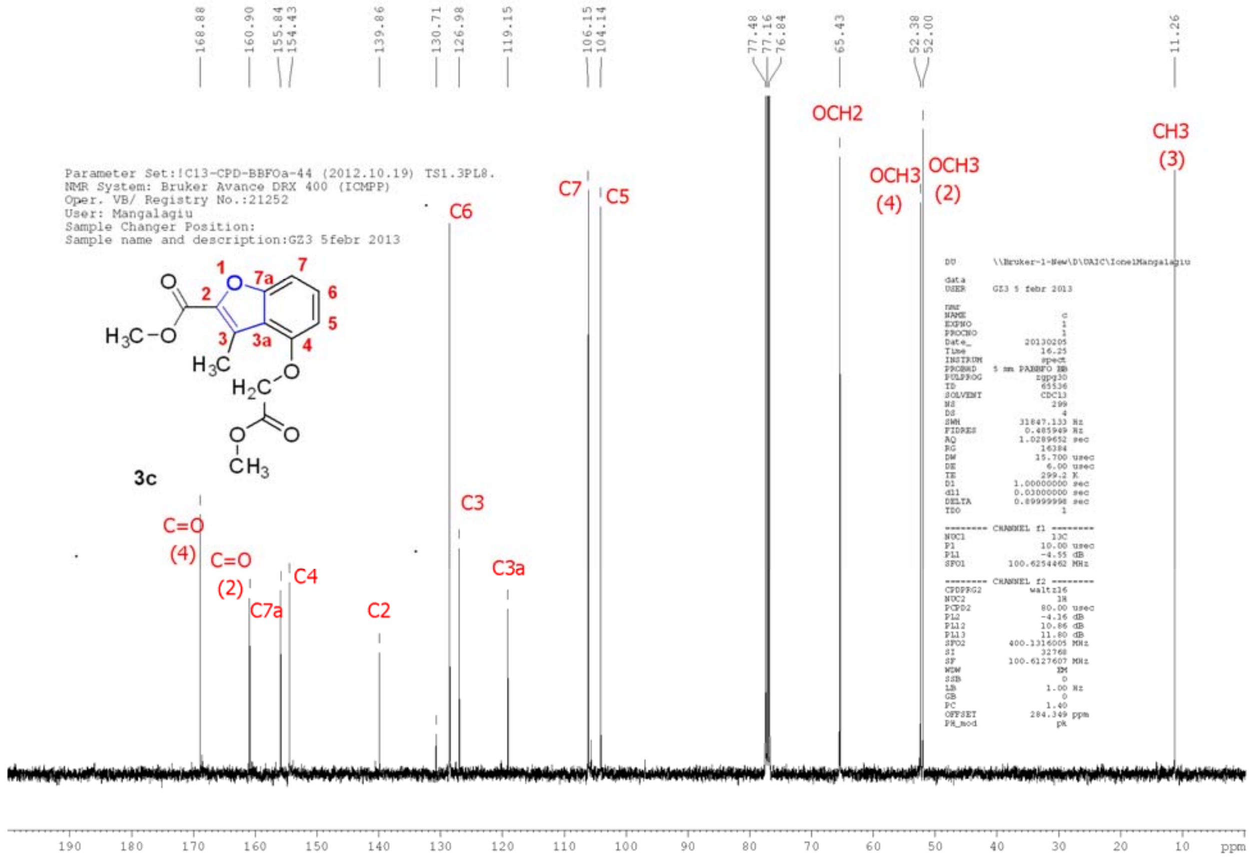


**S9b Fig.** $^{13}$C NMR spectrum of the compound **3c.**

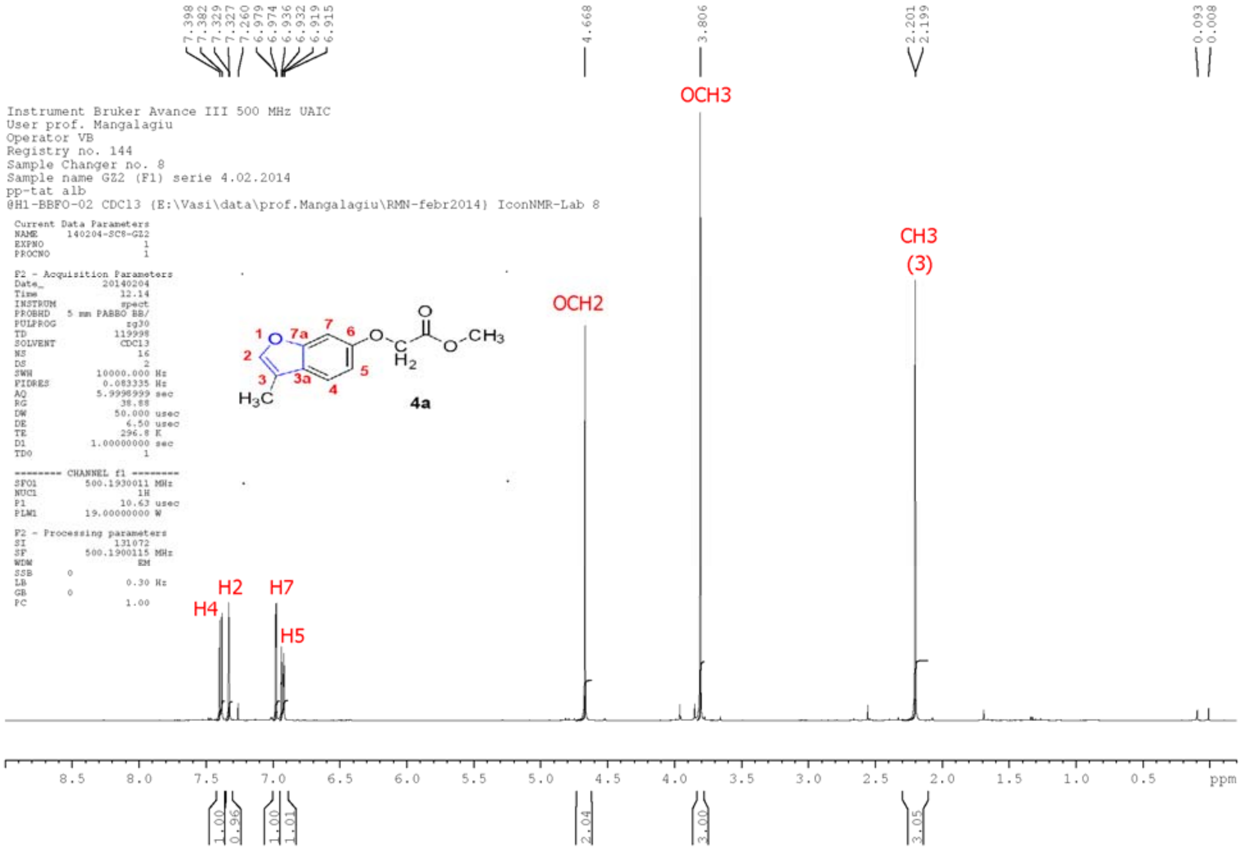


**S10a Fig.** $^{1}$H NMR spectrum of the compound **4a.**

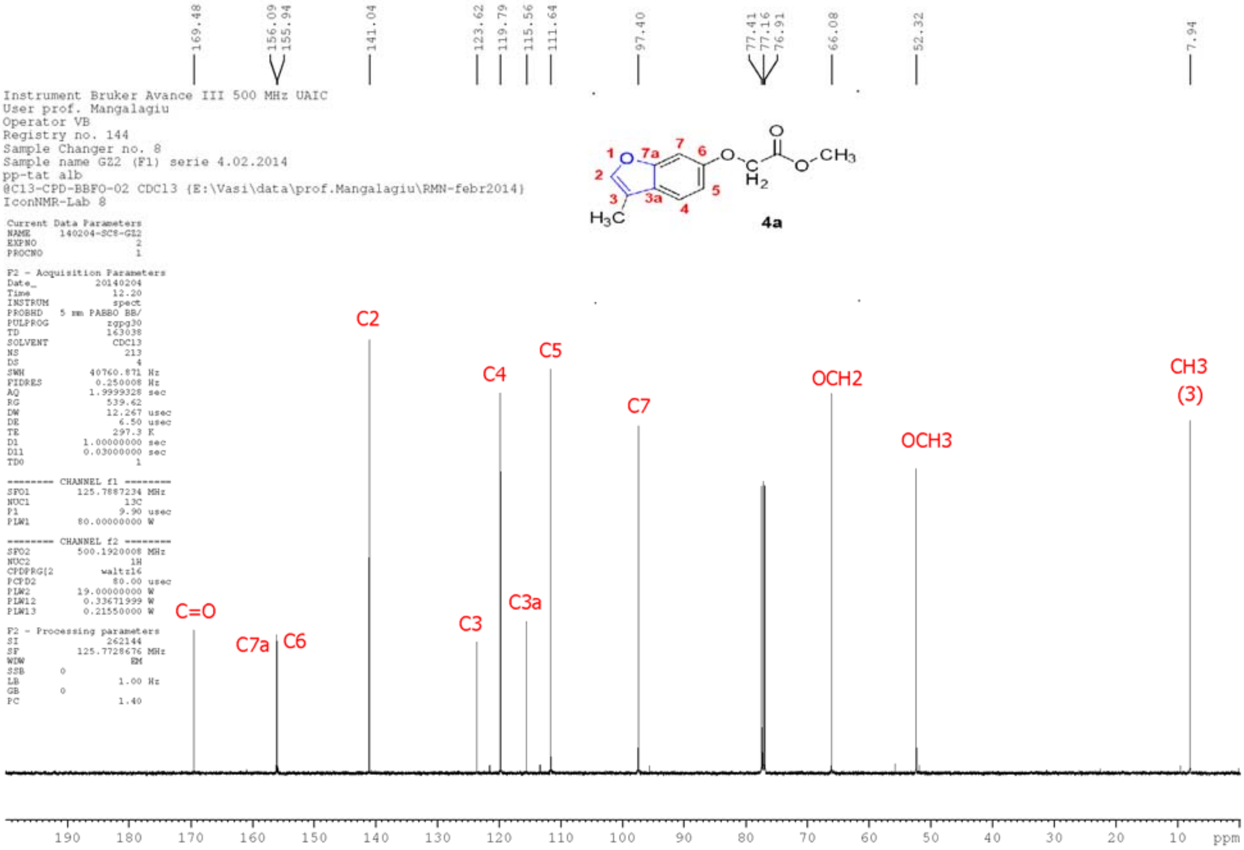


**S10b Fig.** $^{13}$C NMR spectrum of the compound **4a.**

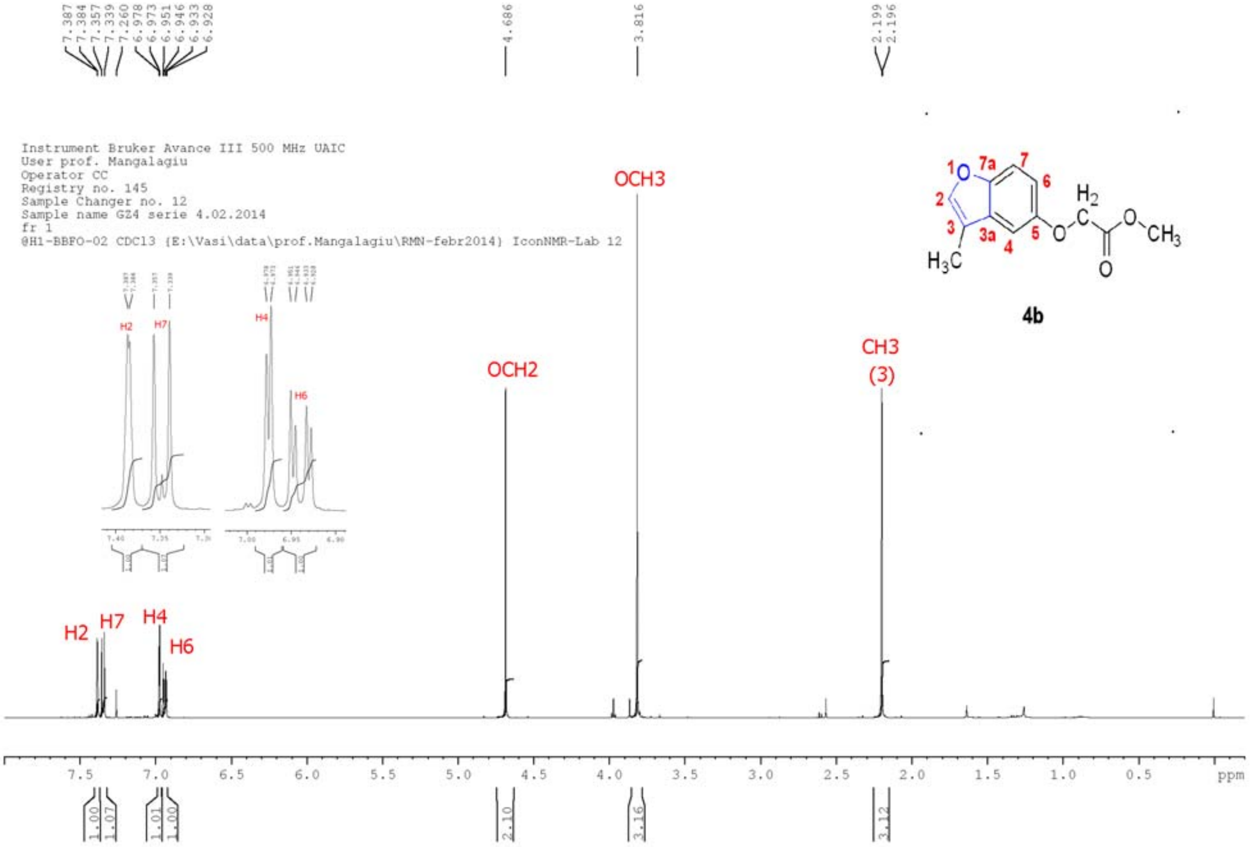


**S11a Fig.** $^{1}$H NMR spectrum of the compound **4b.**

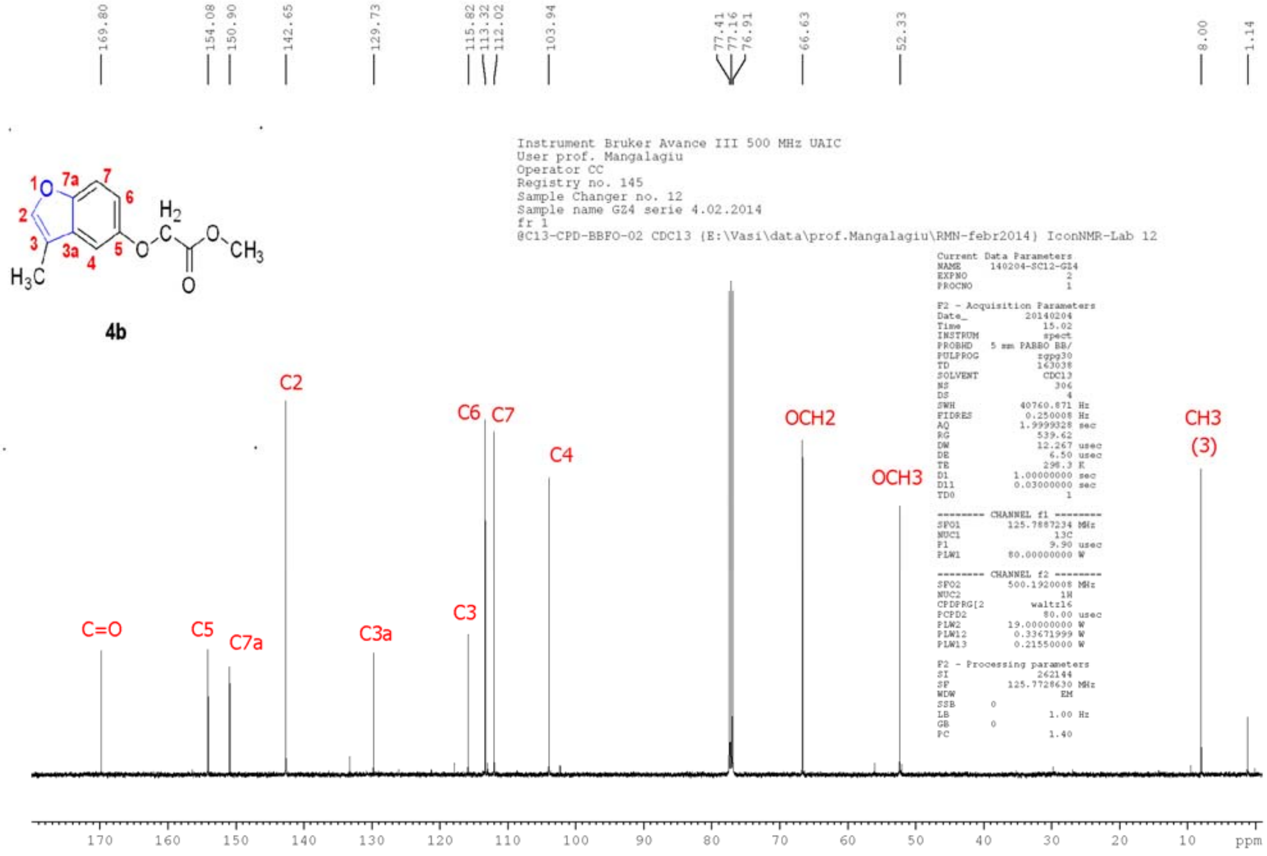


**S11b Fig.** $^{13}$C NMR spectrum of the compound **4b.**

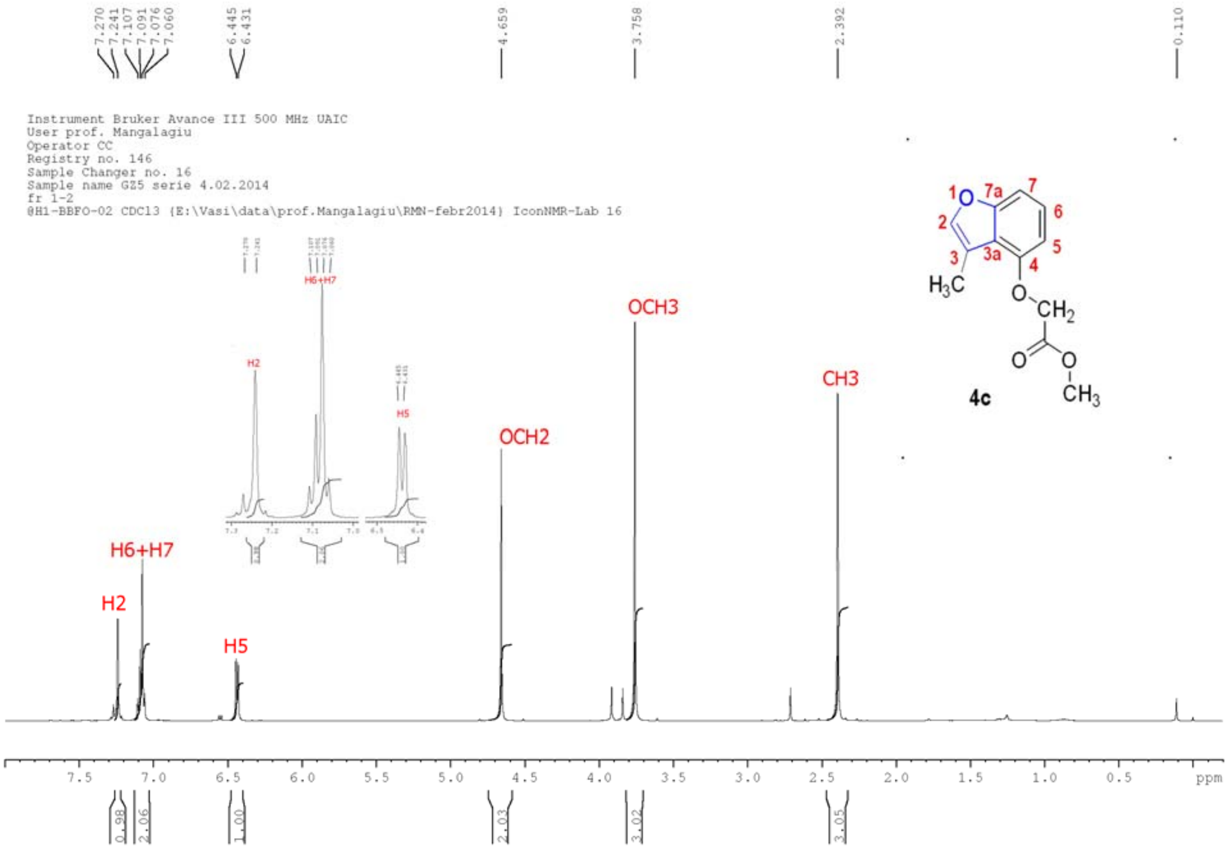

**S12a Fig.** $^{1}$H NMR spectrum of the compound **4c.**

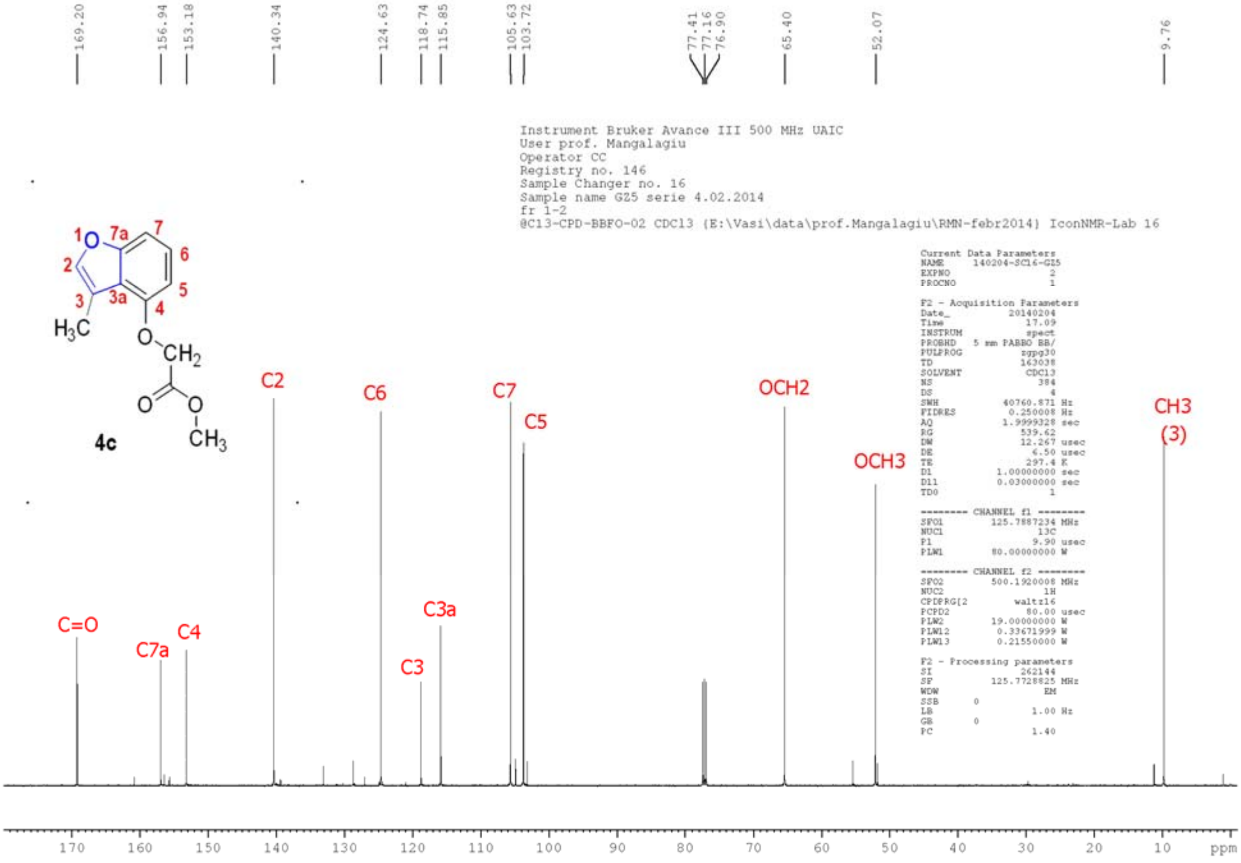

**S12b Fig.** $^{13}$C NMR spectrum of the compound **4c.**

## 3. IR Spectra of the obtained compounds.

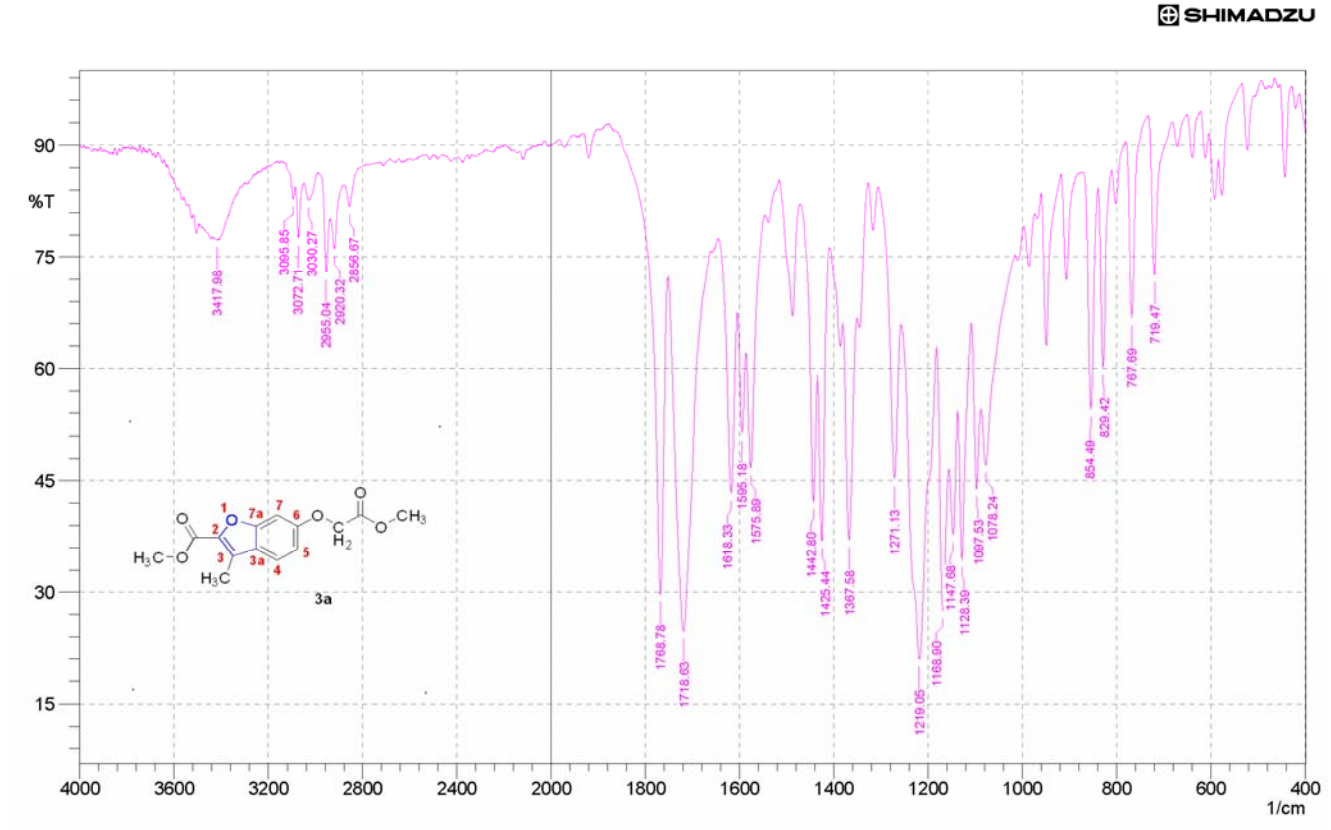


**S13 Fig.** IR spectrum of the compound **3a.**

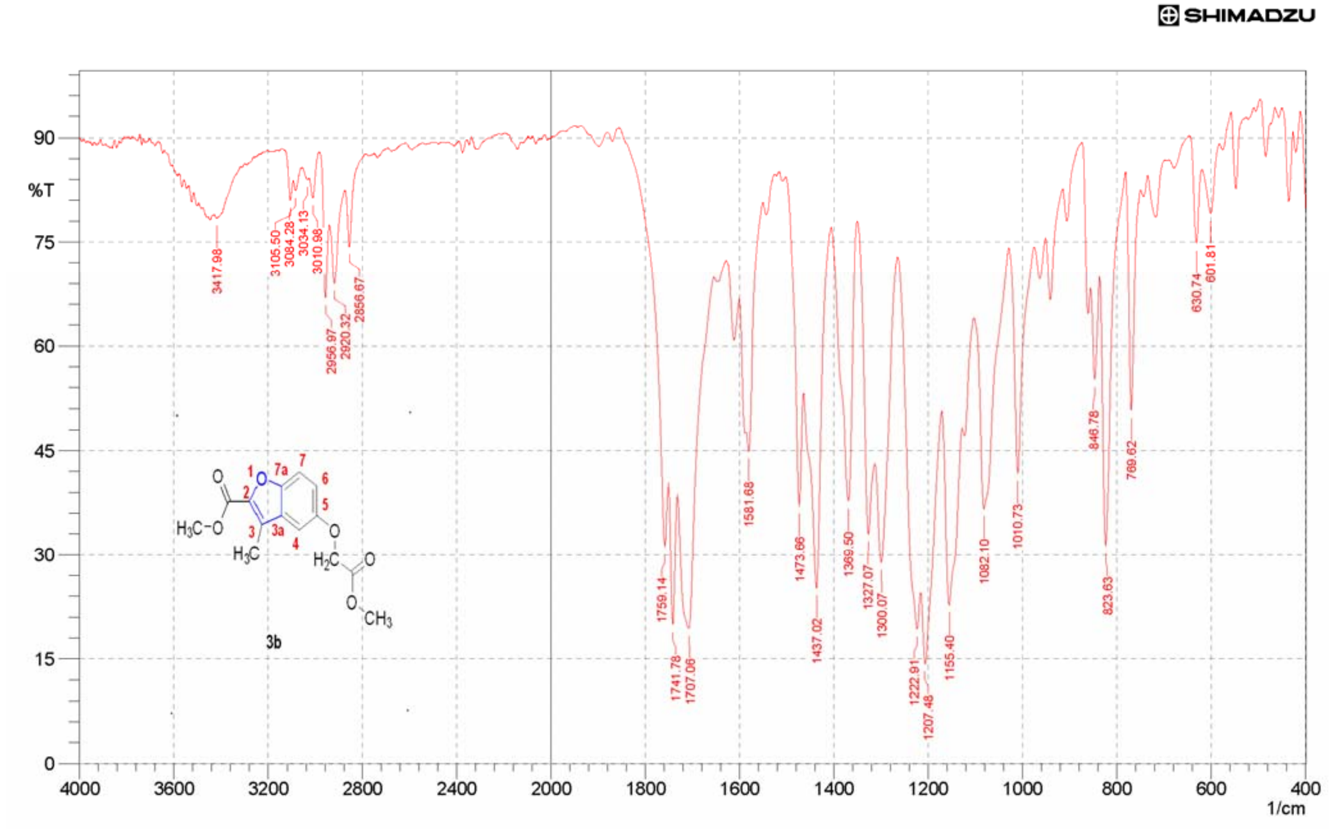


**S14 Fig.** IR spectrum of the compound **3b.**

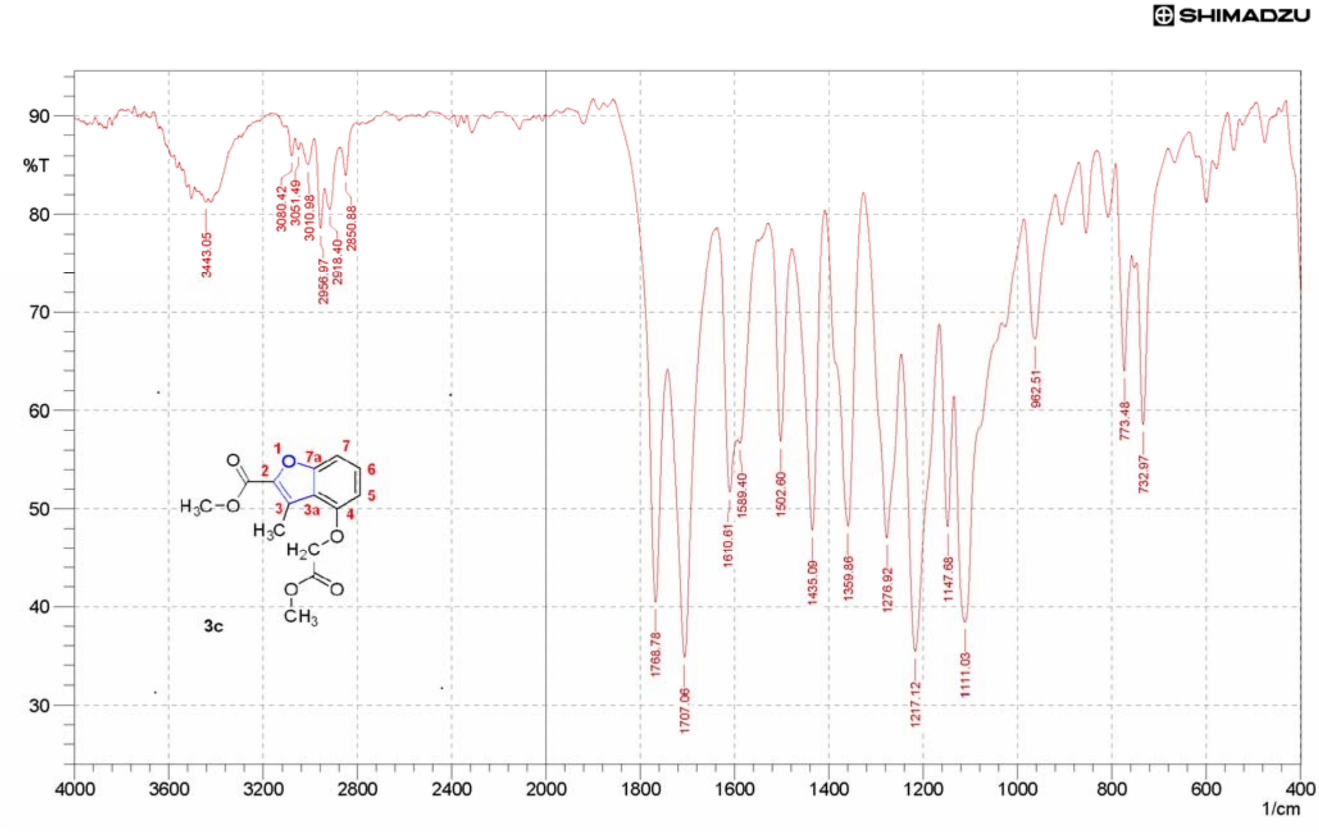


**S15 Fig.** IR spectrum of the compound **3c.**

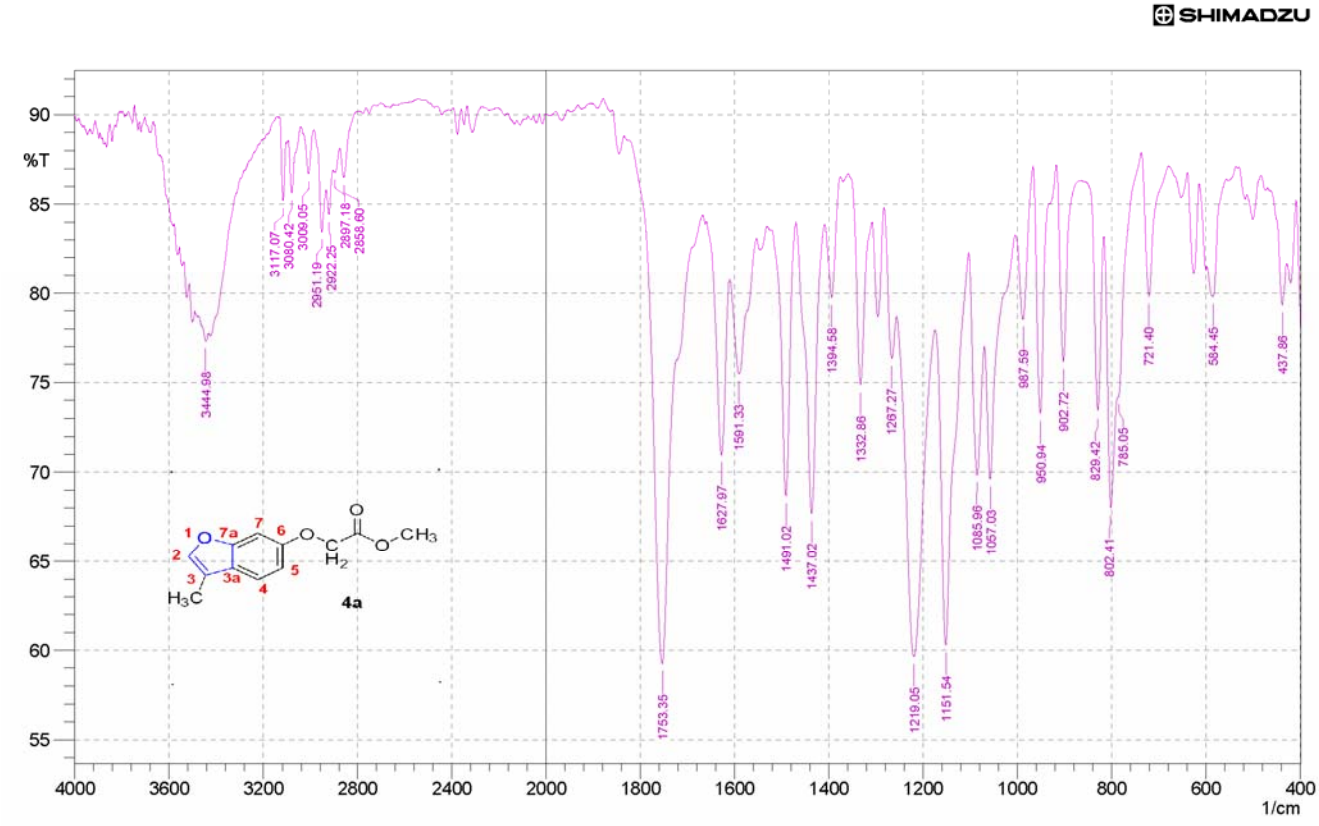


**S16 Fig.** IR spectrum of the compound **4a.**

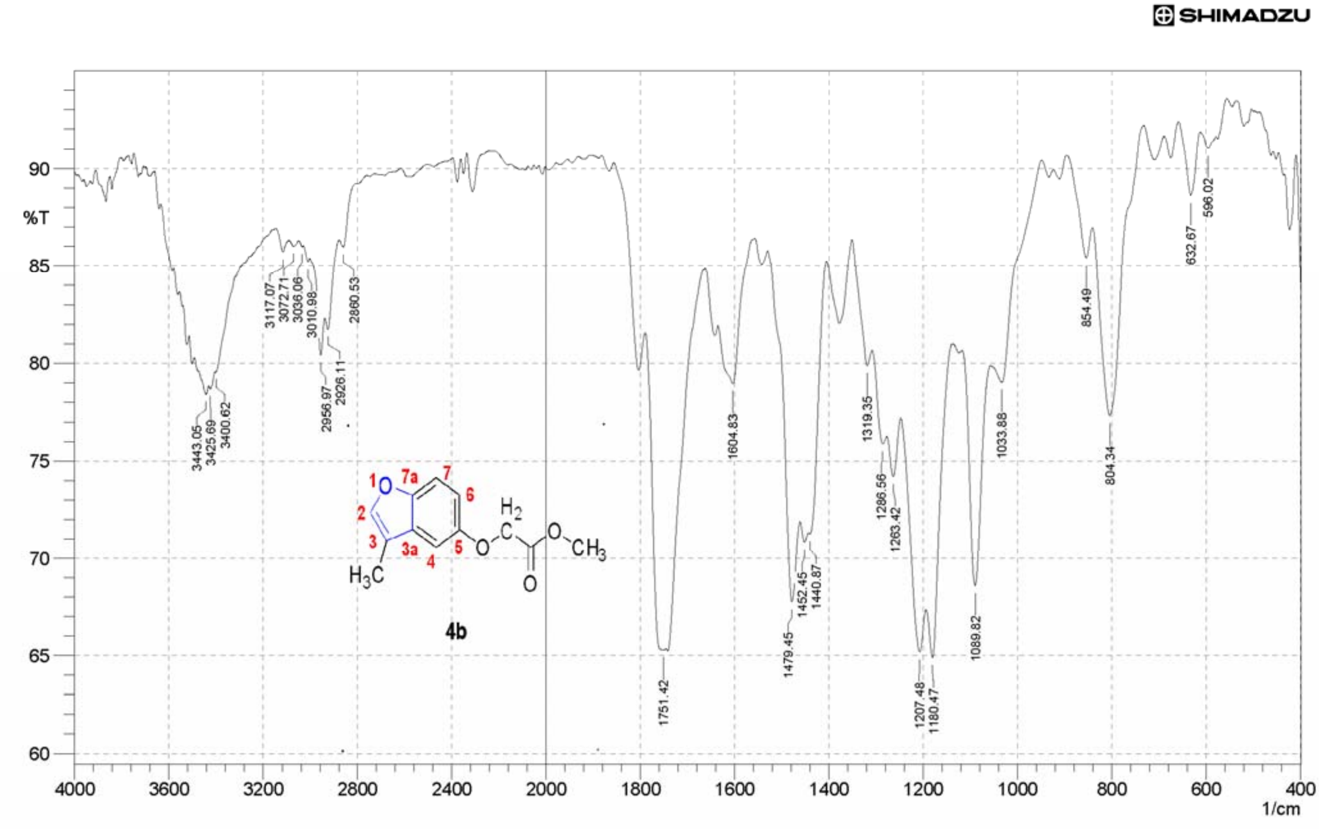

**S17 Fig.** IR spectrum of the compound **4b.**

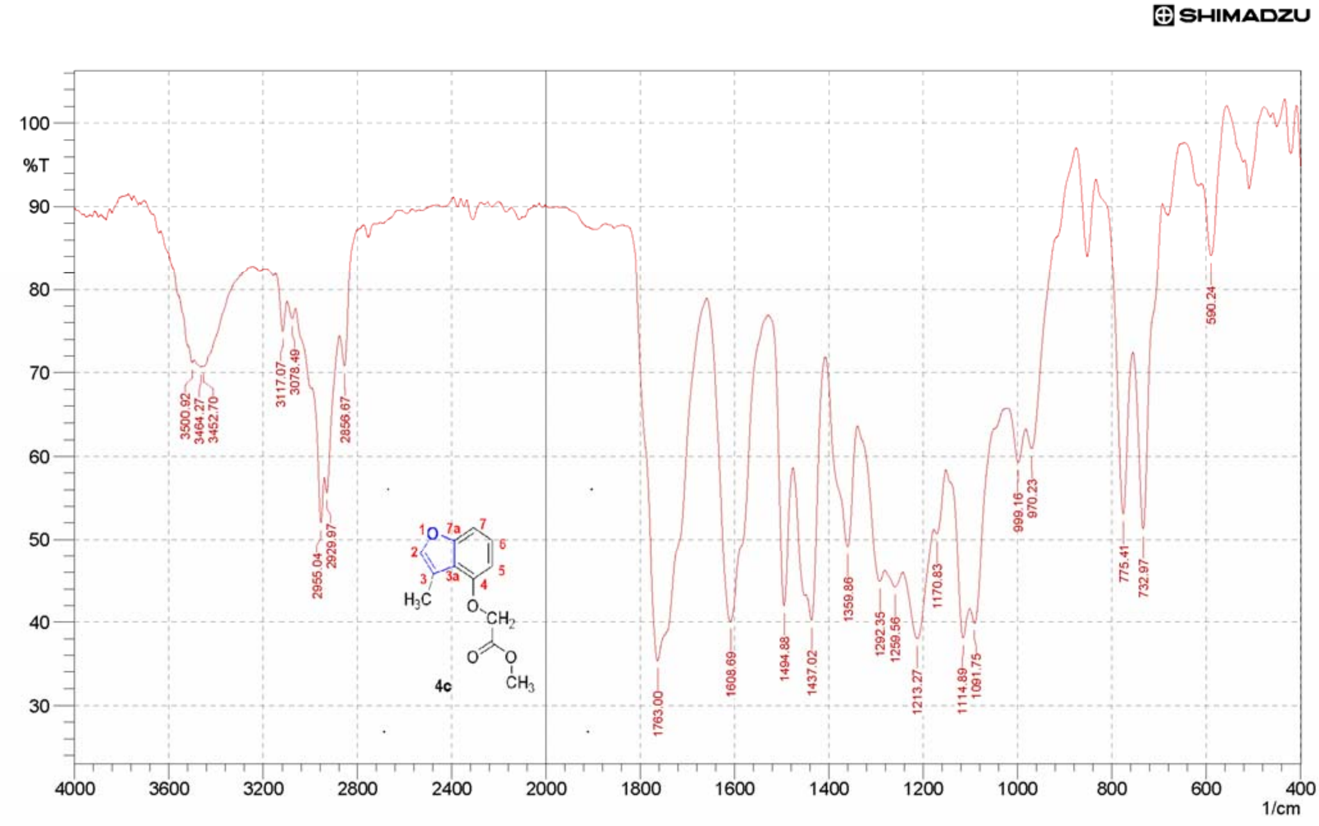

**S18 Fig.** IR spectrum of the compound **4c.**
